# Mutant p53 shapes the enhancer landscape of cancer cells in response to chronic immune signaling

**DOI:** 10.1038/s41467-017-01117-y

**Published:** 2017-09-29

**Authors:** Homa Rahnamoun, Hanbin Lu, Sascha H. Duttke, Christopher Benner, Christopher K. Glass, Shannon M. Lauberth

**Affiliations:** 1 Section of Molecular Biology, University of California, San Diego, 9500 Gilman Drive, La Jolla, CA 92093 USA; 2 Department of Cellular and Molecular Medicine, University of California, San Diego, 9500 Gilman Drive, La Jolla, CA 92093-0651 USA; 3 Department of Medicine, University of California, San Diego, 9500 Gilman Drive, La Jolla, CA 92093-0651 USA

## Abstract

Inflammation influences cancer development, progression, and the efficacy of cancer treatments, yet the mechanisms by which immune signaling drives alterations in the cancer cell transcriptome remain unclear. Using ChIP-seq, RNA-seq, and GRO-seq, here we demonstrate a global overlap in the binding of tumor-promoting p53 mutants and the master proinflammatory regulator NFκB that drives alterations in enhancer and gene activation in response to chronic TNF-α signaling. We show that p53 mutants interact directly with NFκB and that both factors impact the other’s binding at diverse sets of active enhancers. In turn, the simultaneous and cooperative binding of these factors is required to regulate RNAPII recruitment, the synthesis of enhancer RNAs, and the activation of tumor-promoting genes. Collectively, these findings establish a mechanism by which chronic TNF-α signaling orchestrates a functional interplay between mutant p53 and NFκB that underlies altered patterns of cancer-promoting gene expression.

## Abstract (web summary)

Inflammation is known to affect cancer development, yet the mechanisms by which immune signaling drives transformation remain unclear. Here, the authors provide evidence that chronic TNF-α signaling promotes the enhancer binding and transcriptional interplay between mutant p53 and NFκB.

## Introduction

Despite intensive investigation of the crosstalk between tumor and immune cells of the adjacent microenvironment, the mechanisms by which immune signaling drives alterations in the cancer cell transcriptome remain poorly understood. Diffusible immune cell mediators that include chemokines and cytokines function in tumor-promoting inflammation by converging on the activation of transcription factors such as NFκB, which drives altered gene expression programs in cancer cells^1, 2^. Also, the cancer-promoting effects of inflammation vary based on regulatory factors that are linked to genetic aberrations in cancer cells^3^. Of special interest here are *p53* gene mutations, which are the most frequent alterations in human cancer that give rise to mutant proteins that exhibit a loss of their tumor suppressor activities or a gain in their oncogenic (GOF) functions that promote tumorigenesis^4^.

In recent support of the p53 GOF paradigm is the finding that mutant p53 (mutp53) augments NFκB activation and results in chronic but not acute inflammation-induced tumor initiation in a mouse model of inflammatory bowel disease^5^. Another seminal study revealed that mutp53 prolongs NFκB activation by inhibiting apoptosis-stimulated kinase (ASK1)-JNK pathways in response to chronic tumor necrosis factor alpha (TNF-α) signaling^6^. These studies underscore the significance of investigating the mechanisms underlying the interactions between mutp53 and NFκB to advance our understanding of the protumorigenic roles for immune signaling.

Growing evidence supports mutp53 GOF activities that are connected to gene regulation^7, 8^. However, the mechanisms controlling mutp53 DNA binding and transcriptional functions are not well understood. Following our global profiling analyses of mutp53 and NFκB in response to chronic TNF-α signaling, we focus on the possibility of a functional cooperativity between these factors at distinct subsets of enhancers. Enhancers are DNA elements that are activated by transcriptional regulators to orchestrate cell and signal-specific gene expression programs^9–15^. Enhancer activity is often correlated with an enrichment of the histone mark, histone H3 lysine 27 acetylation (H3K27ac)^16–18^ and RNA polymerase II (RNAPII) recruitment that drives the production of bidirectional transcripts known as enhancer RNAs (eRNAs)^19–22^. Despite remarkable progress in understanding the pathways that control enhancer priming and activation, relatively little is understood about the mechanisms that promote enhancer activation for the regulation of oncogenic gene expression programs.

In this study, we uncover enhancers that become active in response to chronic TNF-α signaling as revealed by the global accumulation of the chromatin modifications, histone 3 lysine 4 monomethylation (H3K4me1) and H3K27ac that are hallmarks of active enhancers. In addition, we find that these enhancers integrate mutual mutp53 and NFκB regulation of RNAPII recruitment, the synthesis of eRNAs, and the activation of tumor-promoting genes. Collectively, our findings uncover an enhancer transcription “signature” that is linked to alterations in tumor-promoting gene expression and enhanced cancer cell growth.

## Results

### Alterations in gene expression by TNF signaling and mutp53

**Fig. 1:**
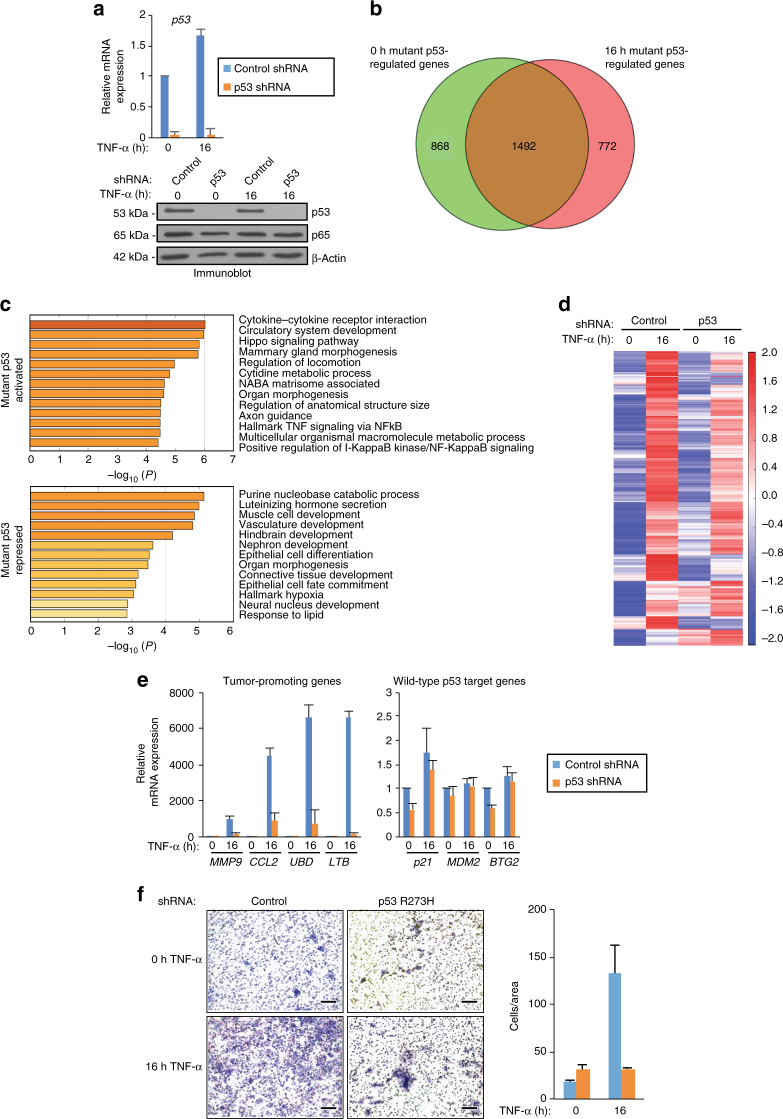
Mutp53 regulates chronic TNF-α induction of protumorgenic genes. **a** qRT-PCR (top) and immunoblot (bottom) analyses of SW480 cells induced to express LacZ (control) or p53 (p53) shRNA and treated with TNF-α for 0 or 16 h. The expression levels following TNF-α treatment are relative to the levels before TNF-α exposure. The bar graph represents the average of three independent experiments with the error bars denoting the standard error. **b** Venn diagram depicting the number of genes affected by mutp53 depletion in SW480 cells treated as described in (**a**). Genes were sorted prior to (green, 0 h) or after (pink, 16 h) TNF-α treatment (FDR < 0.05). **c** Gene Ontology analysis using Metascape of the 772 mutp53-regulated genes upon 16 h TNF-α treatment, corresponding to the pink only portion of the Venn diagram in (**b**). **d** Heat map of the differentially expressed RefSeq genes induced by twofold or higher (FDR < 0.05) after TNF-α induction in the control relative to the mutp53 knockdown cells. **e** qRT-PCR analyses of mutp53 target genes (left) and known wild-type p53 target genes (right) in control and p53 shRNA SW480 cells that were treated with TNF-α for 0 or 16 h. The expression levels shown after TNF-α are relative to the levels before treatment. The bar graph represents the average of three independent experiments with the error bars denoting the standard error. **f** Representative images (left) and quantitation (right) of invasion assays performed with SW480 cells treated as described in (**a**) that were fixed and detected by Giemsa staining (scale bar: 0.2 mm). The bar graph represents the average number of cells invaded through the Matrigel-coated membrane from three independent experiments with the error bars denoting the standard error

To investigate the mechanisms underlying the roles of mutp53 in promoting chronic inflammation-induced tumorigenesis, we performed mRNA sequencing (mRNA-seq) in human SW480 colon cancer cells expressing doxycycline-inducible short hairpin RNAs (shRNAs) against mutp53 before and following TNF-α treatment for 16 h. Relative to a non-targeting shRNA against LacZ (control), p53 shRNA markedly reduced mutp53 R273H mRNA and protein levels before and after TNF-α treatment (+/−TNF-α) (Fig. 1a). By comparing the transcriptomes of control and mutp53-depleted cells (+/−TNF-α), we found that mutp53 alters (≥2-fold, false discovery rate (FDR) < 0.05) a comparable number of genes before (*n* = 2360, 51%) and following (*n* = 2264, 49%) TNF-α treatment (Fig. 1b), which is consistent with a broad role for mutp53 in regulating gene expression. Notably, the colon cancer cell transcriptome regulated by mutp53 is also extensively altered by chronic immune signaling as revealed by the identification of a subset of gained mutp53-regulated genes (*n* = 772) that are upregulated or downregulated in response to TNF-α (Fig. 1b). Analysis of this gained subset of TNF-α-responsive genes revealed 303 (39%) mutp53-repressed genes that include key regulators of organ morphogenesis, development, and hypoxia as revealed by gene ontology (GO) enrichment analysis (Fig. 1c). Conversely, GO categories corresponding to the 469 (61%) mutp53-activated genes are largely related to immunogenic and tumor-promoting processes that include cytokine–cytokine receptor interaction, hallmark TNF signaling via NFκB, and regulation of locomotion (Fig. 1c). Next, we investigated a role for mutp53 in regulating TNF-α-inducible gene expression. As revealed in Fig. 1d and Supplementary Data 1, we identified 482 RefSeq genes that are induced (≥2-fold, FDR < 0.05) by TNF-α in the control knockdown cells. Among the TNF-α-induced genes, we identified a number of genes (*n* = 187, 39%) that are induced in a mutp53-dependent manner (≥2-fold) as revealed by the significant decrease in inducible gene expression following mutp53 depletion (Fig. 1d). Conversely, a much smaller subset (*n* = 9, 2%) of TNF-α-inducible genes are repressed by mutp53 (Fig. 1d). Overall, our mRNA-seq data indicate the role of mutp53 in potent gene activation in response to chronic TNF signaling.

Consistent with our RNA-seq data, quantitative polymerase chain reaction with reverse transcription (qRT-PCR) revealed that mutp53 is required for the induction of a subset of TNF-α-induced genes. Specifically, mutp53 knockdown revealed significant downregulation of the TNF-α-induced expression of *MMP9*, *CCL2*, *UBD*, and *LTB* (Fig. 1e), which are among the top ten most highly induced mutp53-dependent genes identified in our mRNA-seq data (Supplementary Data 1; Fig. 1d). In comparison, mutp53 had little to no effect on the expression of wild-type p53 target genes (Fig. 1e). The TNF-α-inducible gene-selective effects of mutp53 were also identified when using an siRNA oligonucleotide that is directed against a different region of *p53* mRNA in SW480 cells (Supplementary Fig. 1a) and in breast cancer MDA-MB-231 cells that express mutp53 R280K (Supplementary Fig. 1b).

Cell invasion assays were performed to investigate whether TNF-α and mutp53-dependent alterations in the transcriptome are associated with changes in cancer cell growth. As revealed in Fig. 1f, chronic TNF-α results in an approximately sevenfold increase in the number of control cells that pass through the Matrigel. Under uninduced conditions, mutp53 depletion had little to no effect on the number of cells that invade. Notably, however, mutp53 depletion significantly reduced the number of cells that invade following TNF-α signaling for 16 h to a level that is comparable to the number of control cells that invade prior to TNF-α signaling (Fig. 1f). These findings reveal that mutp53 modulates chronic TNF-α-dependent alterations in the cancer cell transcriptome that enhance cancer cell invasion.

### TNF signaling uncovers enhancers occupied by mutp53 and NFκB

**Fig. 2:**
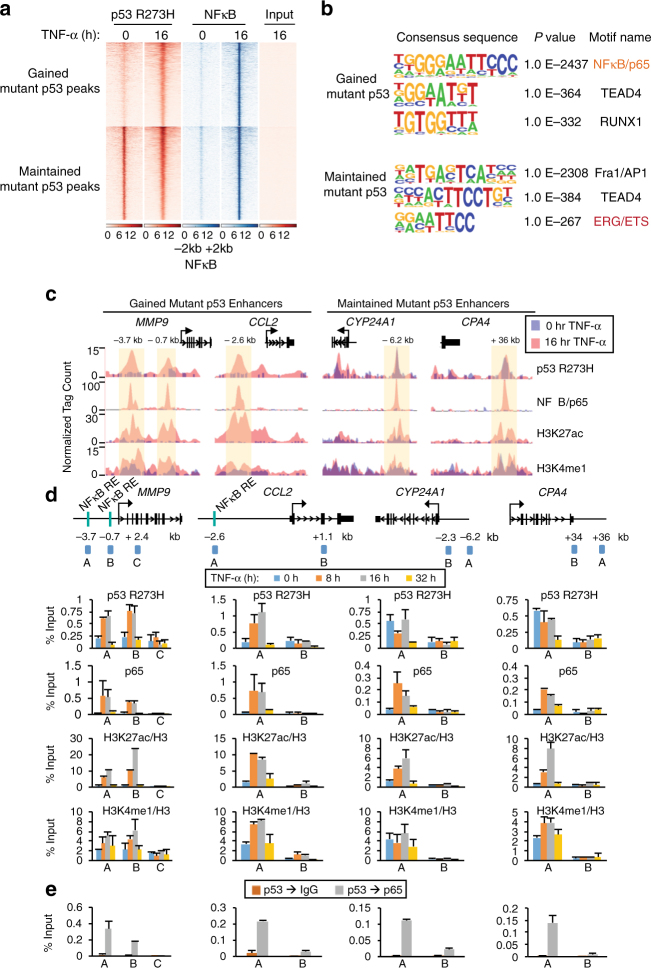
Chronic TNF-α signaling alters mutp53 and NFκB binding in colon cancer cells. **a** Heat maps of p53 R273H and NFκB/p65 ChIP-seq reads in SW480 cells treated with TNF-α for 0 or 16 h. Each row shows ±2 kb centered on p65 peaks, rank-ordered by the intensity of mutp53 and NFκB/p65 peaks and grouped by gained versus maintained mutp53 peaks. **b** De novo motif analyses of the TNF-gained and maintained mutp53 overlapping NFκB/p65 binding sites as noted in (**a**). **c** UCSC genome browser tracks of ChIP-seq signals for p53 R273H, NFκB/p65, H3K27ac, and H3K4me1 at the *MMP9*, *CCL2*, *CYP24A1*, and *CPA4* gene loci in untreated (purple) or TNF-α 16 h (pink)-treated SW480 cells. The *y* axis depicts the ChIP-seq signal and the *x* axis locates the genomic position with the enhancer regions highlighted in yellow. **d** Schematics of ChIP-qPCR amplicons and ChIP analyses with the indicated antibodies at the enhancers and non-specific regions of *MMP9*, *CCL2*, *CYP24A1*, and *CPA4* gene loci. ChIP-qPCR amplicons were designed to amplify the enhancer (A and B at *MMP9* and A at *CCL2*, *CYP24A1*, and *CPA4*) or non-specific (C at *MMP9* and B at *CCL2*, *CYP24A1*, and *CPA4*) regions of the target gene loci. ChIP experiments were performed using SW480 cells treated with TNF-α for 0, 8, 16, and 32 h. ChIPs for histone marks were normalized to H3. An average of two independent ChIP experiments that are representative of at least three is shown with error bars denoting the standard error. **e** Sequential ChIP (re-ChIP) with p53 antibody followed by IgG (control) and NFκB/p65 antibody performed in SW480 cells treated with TNF-α for 16 h. The ChIP-qPCR amplicons are identical to those used in (**d**). An average of two independent re-ChIP experiments that are representative of at least three is shown with error bars denoting the standard error

To further explore the interplay between mutp53 and chronic TNF-α signaling at the genomic level, we performed chromatin immunoprecipitation followed by deep sequencing (ChIP-seq) for mutp53 R273H and NFκB/p65 in SW480 cells (+/−TNF-α). Following TNF-α signaling, stringent p65 peaks (*n* = 24,965; FDR < 0.001) were identified that showed a striking colocalization with unchanged or “maintained” mutp53 peaks (*n* = 18,295; FDR < 0.001) and TNF-α-dependent enriched or “gained” mutp53 peaks (*n* = 17,772; FDR < 0.001) (Fig. 2a). Specifically, 43% of the gained mutp53-binding sites show overlap with p65, and conversely 31% of the p65-occupied regions overlap with the gained mutp53 peaks in response to TNF-α (Supplementary Fig. 2a). In addition, de novo motif analysis identified that NFκB consensus motifs are among the most highly enriched to overlap with the gained mutp53 and NFκB peaks (Fig. 2b). We also identified a comparable degree of overlap between p65 and maintained mutp53-binding sites following TNF-α exposure with about half (47%) of the maintained mutp53 regions showing overlap with p65 and 34% of the p65-enriched regions revealing overlap with maintained mutp53-binding sites (Fig. 2a; Supplementary Fig. 2a). Motif analysis of the maintained peaks revealed an enrichment of the consensus sequences recognized by transcription factors that include Fra-1/AP-1, TEAD4, and ERG/ETS but not NFκB (Fig. 2b). Also as expected, wild-type p53 motifs do not overlap with the gained or maintained mutp53 peaks, which is consistent with the reduced affinity of contact mutp53 for wild-type p53 recognition elements^23^. Altogether, these findings reveal that chronic TNF-α promotes an overlap in NFκB and mutp53 binding across the genome.

Genome-wide analyses have revealed that the majority of NFκB binding takes place at genomic regions with enhancer-like chromatin features^9, 24^. To explore whether the overlapping mutp53 and NFκB-binding sites occur at enhancers, we compared our mutp53 and NFκB ChIP-seq data to global profiling analysis of H3K27ac and H3K4me1 that demarcate enhancers. As demonstrated at the gained (*MMP9* and *CCL2*) and maintained (*CYP24A1* and *CPA4*) mutp53-bound enhancers, we identified a strong correlation between mutp53 and NFκB enrichment, and H3K4me1 and H3K27ac accumulation that occurs across the genome in response to chronic TNF-α signaling (Fig. 2c).

To determine the temporal relationship of mutp53 and NFκB binding at the identified enhancers, we next performed time course ChIP experiments followed by qPCR. Immunoprecipitated DNA was analyzed with primer sets specific to regions with active enhancer chromatin signatures and centered at NFκB/p65 and mutp53 peaks (Fig. 2d, amplicons A and B at *MMP9* and A at *CCL2*, *CYP24A1*, and *CPA4*) and non-specific control regions (Fig. 2d, amplicon C at *MMP9* and B at *CCL2*, *CYP24A1*, and *CPA4*). Consistent with our ChIP-seq data, ChIP-qPCR revealed low levels of pre-associated mutp53 and negligible NFκB binding before TNF-α signaling and substantial TNF-α-induced increases in the binding of both factors at the gained enhancers, but not at the control regions of the *MMP9* and *CCL2* genes (Fig. 2d). At the maintained enhancers, *CYP24A1* and *CPA4*, we observed comparable levels of mutp53 binding +/−TNF-α, and negligible levels of uninduced NFκB binding that are increased in response to TNF-α (Fig. 2d). Consistent with our ChIP-seq findings, H3K4me1 and H3K27ac levels were observed at the mutp53 and NFκB co-bound enhancers with peak levels observed at 8 and 16 h TNF-α (Fig. 2d). In addition, the overlap of mutp53, NFκB, and H3K27ac that occurs in response to chronic TNF-α was identified at the *MMP9* and *CPA4* enhancer regions, but not the wild-type p53 *p21* enhancer in MDA-MB-231 cells (Supplementary Fig. 2b). Similarly, wild-type p53 was not found to overlap with NFκB or H3K27ac at the *MMP9* or *CPA4* enhancers in HCT116 cells, despite the enrichment of wild-type p53 at the *p21* enhancer (Supplementary Fig. 2c). These ChIP analyses support differential enhancer targeting of mutant versus wild-type p53 in cancer cells in response to chronic immune signaling.

To investigate simultaneous binding of mutp53 and NFκB at active enhancers, sequential chromatin immunoprecipitation (re-ChIP) for mutp53 followed by NFκB was performed in SW480 cells treated with TNF-α for 16 h. As shown in Fig. 2e, qPCR analysis of the re-ChIP DNA revealed NFκB and mutp53 co-occupancy at the *MMP9*, *CCL2*, *CYP24A1*, and *CPA4* enhancers. In comparison, the control (IgG) IP revealed little to no enrichment of DNA in the re-ChIP experiments. The strong parallel between our global and enhancer-specific analyses demonstrate distinct classes of mutp53 and NFκB co-bound enhancers.

### Mutp53 and NFκB form functional interactions

**Fig. 3:**
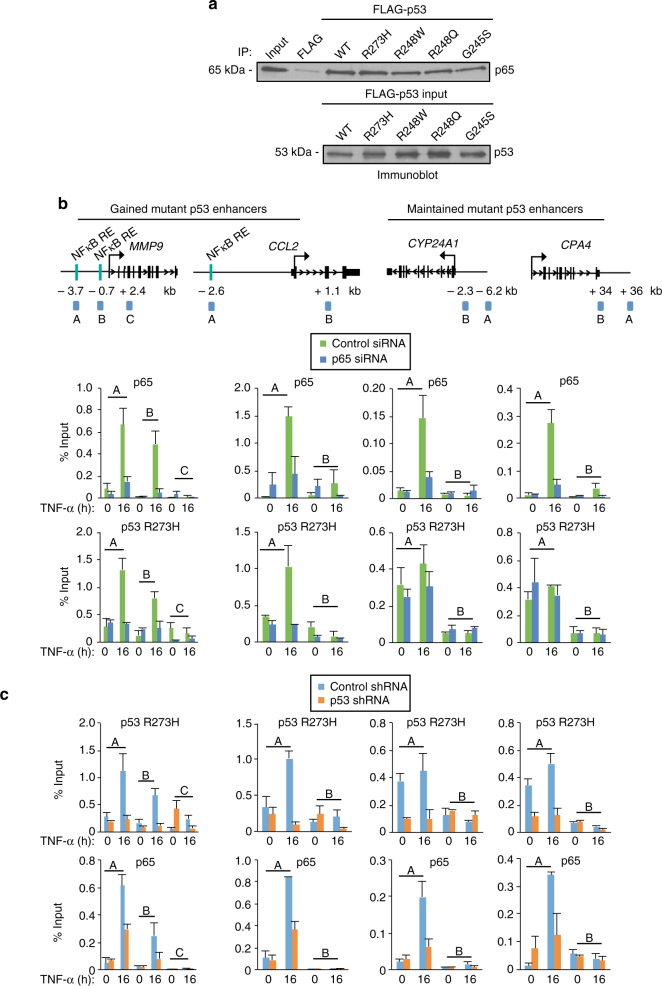
Mutp53 and NFκB interact and impact each other’s binding at enhancers. **a** Purified wild-type and mutp53 proteins bind directly to purified NFκB/p65 as revealed by immunoblot analysis with an antibody that recognizes p65. Input samples for the p53 proteins were also analyzed by immunoblot analysis with an antibody that recognizes both wild-type and mutp53. Three independent interaction assays were performed. **b** ChIP-qPCR analyses of NFκB/p65 and p53 R273H at the enhancer (A and B at *MMP9* and A at *CCL2*, *CYP24A1*, and *CPA4*) or non-specific (C at *MMP9* and B at *CCL2*, *CYP24A1*, and *CPA4*) regions of the target gene loci in SW480 cells transfected with non-targeting control or p65 siRNA and following TNF-α treatment for 0 or 16 h. **c** ChIP-qPCR analyses of p53 R273H and NFκB/p65 binding at identical genomic regions examined in (**b**). The SW480 cells were induced to express LacZ (control) or p53 (p53) shRNA and treated with TNF-α for 0 or 16 h. For both ChIP experiments, an average of two independent experiments that are representative of at least three is shown with error bars denoting the standard error

Given the striking overlap of the mutp53 and NFκB-binding profiles, we next assessed whether these factors form functional interactions. Using purified mutp53 and p65 proteins (Supplementary Fig. 3a), we identified that p65 interacts directly and equivalently with the p53 mutants, R273H, R248W, R248Q, and G245S (Fig. 3a). p65 also interacts directly with wild-type p53 (Fig. 3a), despite that these factors were not found to overlap at mutp53-bound enhancers (Supplementary Fig. 2c). As shown in Supplementary Fig. 3b, we also identified an association between mutp53 and NFκB in SW480 cells. Specifically, an NFκB/p65 antibody comparably co-immunoprecipitated mutp53 from nuclear extracts prepared before and following TNF-α treatment. These results establish mutp53–NFκB interactions that are consistent with the TNF-α-induced global overlap (Fig. 2a, c; Supplementary Fig. 2a) and the simultaneous binding of mutp53 and NFκB at active enhancers (Figs. 2d, e).

We next analyzed whether NFκB regulates mutp53 enhancer binding. ChIP-qPCR was performed in SW480 cells transfected with control or NFκB/p65 siRNA, which reduced NFκB mRNA and protein levels without affecting mutp53 levels (Supplementary Fig. 3c). Under TNF-α-induced conditions, p65 knockdown resulted in a comparable loss of p65 binding at the *MMP9* (80%, 90% at amplicons A and B, respectively) and *CCL2* (70%) enhancers (Fig. 3b). Notably, decreased p65 binding resulted in a substantial and comparable reduction of mutp53 binding at the *MMP9* (70%, 75% at amplicons A and B, respectively) and *CCL2* (77%) enhancers (Fig. 3b). In comparison, little to no effect of p65 knockdown on mutp53 binding was identified at the maintained mutp53 enhancers, *CYP24A1* and *CPA4*, despite the significant decrease (73% and 83%, respectively) in the TNF-α-induced levels of NFκB binding at both enhancers (Fig. 3b). This finding is consistent with our motif analysis (Fig. 2b), which revealed that NFκB response elements are not enriched, whereas response elements recognized by transcription factors including ETS2 overlap with the maintained enhancers. Also consistent with the motif analysis (Fig. 2b), we identified a comparable enrichment of ETS2 that overlaps with mutp53 binding at the maintained enhancers, *CYP24A1* and *CPA4* before and after TNF-α signaling (Supplementary Fig. 3d), which is consistent with published results showing that ETS2 facilitates mutp53 binding^25, 26^. We further investigated the role of NFκB in regulating mutp53 binding at the gained enhancers, *MMP9* and *CCL2* by using the proteasome inhibitor MG132, which prevents degradation of IκB, an endogenous NFκB inhibitor^27^, while not affecting the protein levels of p65 or mutp53 (Supplementary Fig. 3e). Consistent with the NFκB knockdown experiments (Fig. 3b), MG132 significantly reduced p65 binding at the *MMP9* (96%) and *CCL2* (89%) enhancers (Supplementary Fig. 3f), which resulted in substantial losses of TNF-induced mutp53 binding at the *MMP9* (86%, 94% at amplicons A and B, respectively) and *CCL2* (88%) enhancers (Supplementary Fig. 3f). Altogether, these results demonstrate that NFκB is required for mutp53 binding at a subset of enhancers in response to TNF-α signaling.

We next investigated a role for mutp53 in the regulation of NFκB enhancer binding by performing ChIP-qPCR in SW480 cells following mutp53 shRNA and siRNA-mediated knockdown. As shown in Fig. 1a, mutp53 knockdown significantly decreased mutp53 mRNA and protein levels, without affecting p65 levels. Under uninduced conditions, mutp53 binding at the maintained, *CYP24A1* (74%) and *CPA4* (66%) enhancers were significantly decreased following mutp53 knockdown, which is consistent with the identification of pre-associated mutp53 binding at this subset of enhancers (Fig. 3c). In TNF-α-treated cells, mutp53 knockdown resulted in a comparable and substantial decrease in mutp53 binding at all four enhancers, *MMP9* (80%, 82% at amplicons A and B, respectively), *CCL2* (91%), *CYP24A1* (78%), and *CPA4* (75%) (Fig. 3c), which resulted in a significant decrease in p65 binding at the *MMP9* (50%, 67% at amplicons A and B, respectively), *CCL2* (57%), *CYP24A1* (63%), and *CPA4* (68%) enhancers (Fig. 3c). The effect of mutp53 in regulating p65 binding at the maintained enhancers that are devoid of NFκB consensus motifs (Fig. 2b) is consistent with a mutp53 GOF activity that involves altering NFκB binding to extend the cancer cell transcriptome. The requirement for mutp53 in regulating NFκB recruitment was confirmed using siRNA-mediated knockdown of mutp53 (Supplementary Fig. 3g). The ability of mutp53 and NFκB to impact each other’s binding is in agreement with the identification of mutp53–NFκB interactions and the interplay of these factors at specific subsets of enhancers in response to chronic TNF-α signaling.

### Potent eRNA synthesis at mutp53 and NFκB co-bound enhancers

**Fig. 4:**
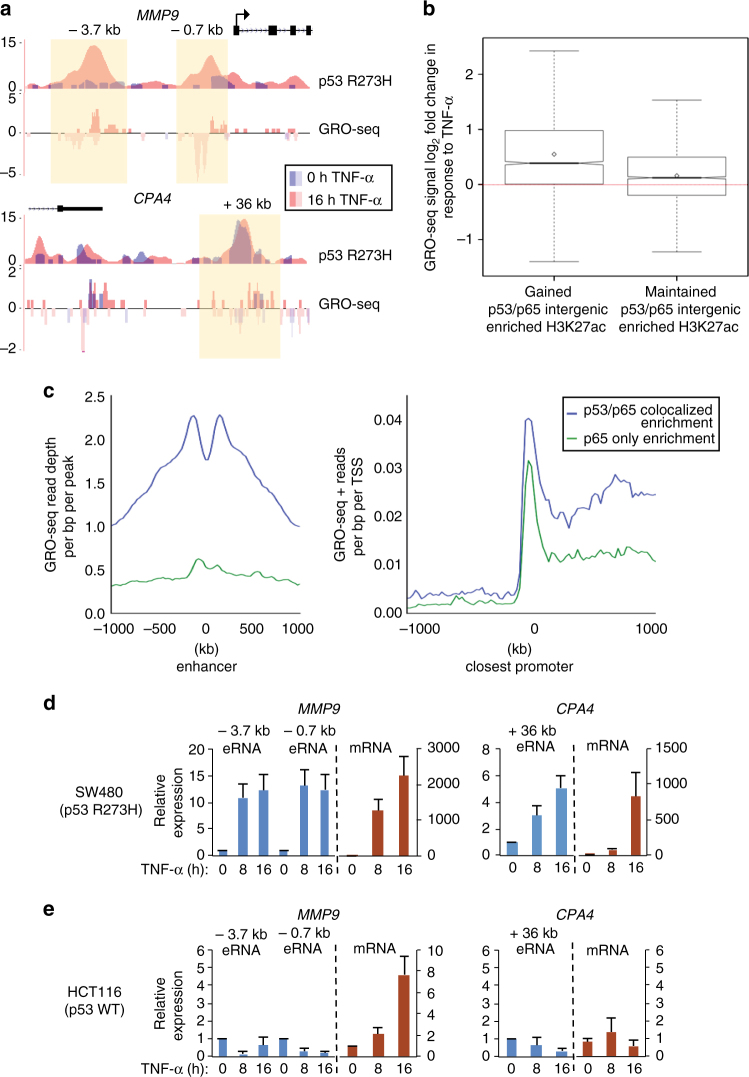
Mutp53 enhancer binding is positively correlated with enhancer transcription. **a** UCSC genome browser images for *MMP9* and *CPA4* gene loci showing the ChIP-seq signal for mutp53 binding and GRO-seq peaks with the enhancer regions highlighted in yellow. **b** Boxplots showing log_2_ fold change in response to TNF-α in GRO-seq signal of nascent transcripts centered upon intergenic gained or maintained mutp53 peaks that overlap with NFκB and H3K27ac peaks. **c** Analyses of (left) GRO-seq reads per bp per intergenic ChIP-seq peaks of mutp53 and NFκB/p65, as indicated, and (right) GRO-seq reads per bp per TSS at promoters closest to intergenic ChIP-seq peaks defined in the left panel. **d** qRT-PCR analyses of the indicated eRNAs and mRNAs in SW480 cells and **e** HCT116 cells treated with TNF-α for 0, 8, or 16 h. The expression levels shown after TNF-α treatment are relative to the levels before treatment. The bar graphs represent the average of three independent experiments with the error bars denoting the standard error

Given that active enhancers support eRNA synthesis, we performed global run-on sequencing (GRO-seq) to assay nascent transcription in SW480 cells (+/−TNF-α). As demonstrated at the *MMP9* and *CPA4* enhancers, our GRO-seq data reveals bidirectional transcription at mutp53 and NFκB co-bound enhancers (Fig. 4a). Specifically, the gained *MMP9* enhancer revealed low levels of uninduced and markedly induced eRNA levels following TNF-α treatment, which parallels with the induced levels of mutp53 binding (Fig. 4a). In comparison, the maintained *CPA4* enhancer supports comparable levels of eRNA synthesis that parallels with mutp53 binding before and following TNF-α (Fig. 4a). This correlation between eRNA synthesis and mutp53 binding was further demonstrated at the *CCL2* and *CYP24A1* enhancers, respectively (Supplementary Fig. 4a). To further investigate differential eRNA induction from mutp53 gained and maintained enhancers, we examined the GRO-seq signals from all enhancers parsed by mutp53, NFκB, and H3K27ac enrichment. The vast majority of the mutp53 gained (*n* = 1506, 58%) and maintained (*n* = 1864, 68%) enhancers reveal robust TNF-α-inducible eRNA synthesis (Fig. 4b). In addition, the higher levels of uninduced eRNA synthesis from the maintained enhancers is consistent with the lower fold eRNA induction that is identified from the maintained versus gained enhancers (Fig. 4b). These results, taken together with the identification of H3K27ac accumulation are consistent with an active state of the mutp53 and NFκB co-bound enhancers in response to chronic TNF signaling. These findings also reveal a strong correlation between mutp53 enhancer localization and enhancer-directed transcription across the genome.

We next wanted to determine whether enhancer transcription at mutp53 and NFκB co-bound enhancers parallels with the transcription of nearby genes on a global scale. Thus, we compared our GRO-seq data at intergenic sites bound by NFκB alone or co-bound by mutp53 and NFκB with the positive strand GRO-seq signals identified at the nearest gene promoters following TNF-α treatment. Notably, the peak GRO-seq signals were significantly higher at the mutp53 and NFκB co-bound enhancers as compared to the NFκB-bound enhancers (Fig. 4c, left). Also, there exists a strong parallel between intergenic GRO-seq signals at the two subsets of enhancers and the GRO-seq signals at the nearby promoters (Fig. 4c, right). These results indicate that enhancer transcription positively correlates with mutp53 binding and nearby gene activation.

To further investigate the kinetics of enhancer transcription from mutp53 and NFκB co-bound enhancers, eRNA levels were examined in SW480 cells treated with TNF-α for 0, 8, and 16 h. We identified that eRNA induction reached near maximal levels from the maintained (*CYP24A1* and *CPA4*) and gained (*MMP9* and *CCL2*) enhancers by 8 h of TNF-α treatment (Fig. 4d; Supplementary Fig. 4b). Consistent with our GRO-seq data (Fig. 4a–c), the overall fold induction of eRNA levels are (three- to fourfold) higher at the gained versus maintained mutp53-bound enhancers, which is due to the measurable levels of eRNA synthesis at the maintained but not gained enhancers in uninduced cells (Fig. 4d; Supplementary Fig. 4b). The strong correlation between enhancer transcription and nearby-gene activation was further confirmed by qRT-PCR analyses of the mRNA expression levels of the *MMP9*, *CCL2*, *CYP24A1*, and *CPA4* genes (Fig. 4d; Supplementary Fig. 4b). In comparison, negligible levels of eRNA synthesis were identified from the *MMP9*, *CCL2*, *CYP24A1*, and *CPA4* enhancers in wild-type p53-expressing HCT116 cells, which is consistent with the negligible TNF-α-induced mRNA expression levels of all four genes (Fig. 4e; Supplementary Fig. 4c). A 250-fold lower level of TNF-α-induced *MMP9* expression was detected in HCT116 as compared to SW480 cells through a mechanism that is independent of mutp53 (Fig. 4e; Supplementary Fig. 4c). These enhancer-specific findings and our genome-wide data provide support for a functional interplay between mutp53 enhancer-binding sites, enhancer transcription, and target gene expression.

### Mutp53 and NFκB regulate RNAPII binding and eRNA production

**Fig. 5:**
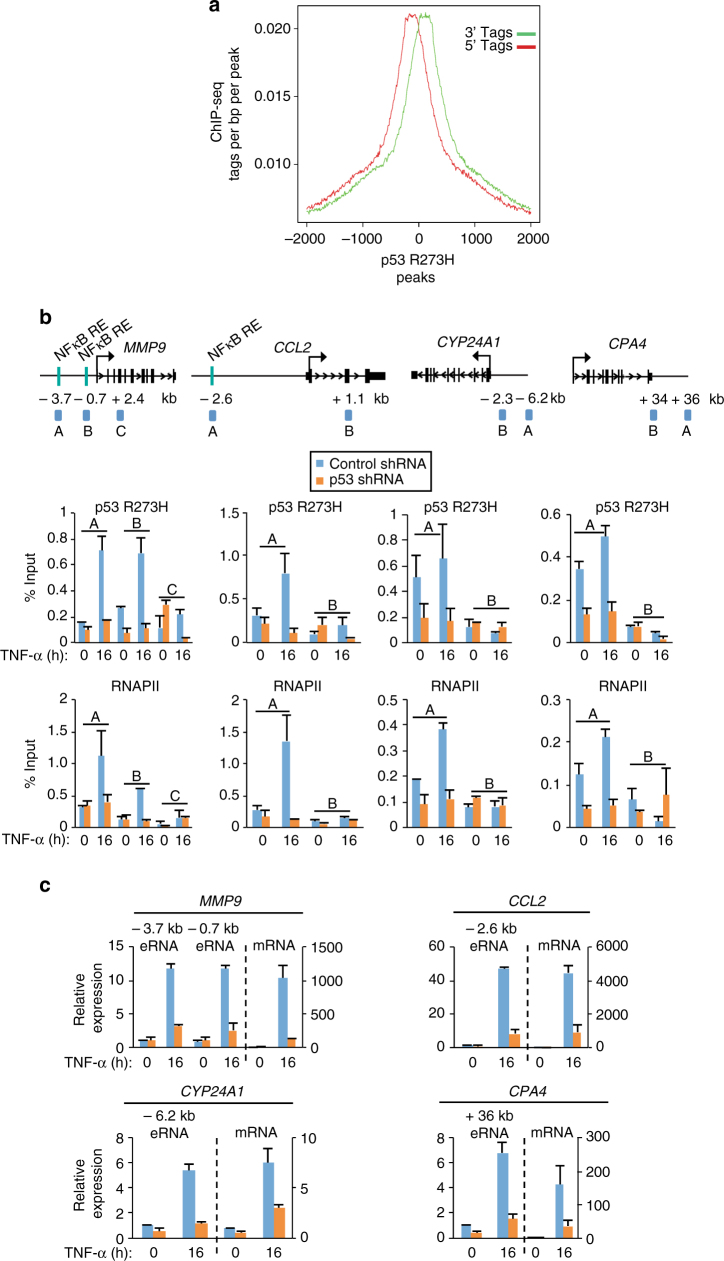
Mutp53 regulates RNAPII recruitment and enhancer transcription. **a** RNAPII ChIP-seq peaks overlapping with mutp53 peaks following 16 h TNF-α treatment. **b** ChIP-qPCR analyses of p53 R273H and RNAPII enrichment at *MMP9*, *CCL2*, *CYP24A1*, and *CPA4* enhancers in SW480 cells expressing control (LacZ) or p53 shRNA and treated with TNF-α for 0 or 16 h. An average of two independent ChIP experiments that are representative of at least three is shown with error bars denoting the standard error. The amplicons used for ChIP-qPCR are shown in the schematics and were designed to recognize the enhancer (A and B at *MMP9* and A at *CCL2*, *CYP24A1*, and *CPA4*) or non-specific (C at *MMP9* and B at *CCL2*, *CYP24A1*, and *CPA4*) regions of the target gene loci. **c** qRT-PCR analyses of the indicated eRNAs and mRNAs in SW480 cells treated as described in (**b**). The expression levels shown after TNF-α treatment are relative to the levels before treatment. The bar graphs represent the average of three independent experiments with the error bars denoting the standard error

We next investigated a direct role for mutp53 in the regulation of eRNA synthesis. First, using ChIP-seq we compared the global-binding profiles of RNAPII and mutp53, which revealed a significant colocalization of RNAPII and mutp53 in response to TNF-α signaling (Fig. 5a), and a symmetrical and bidirectional profile for global RNAPII binding (Fig. 5a) that is consistent with bidirectional transcription from the mutp53-bound enhancers (Figs 4a–c; Supplementary Fig. 4a). Next, we examined a role for mutp53 in the regulation of RNAPII binding at mutp53-bound enhancers by performing RNAPII ChIP following the inducible expression of control or mutp53 shRNA. Under uninduced conditions, negligible levels of mutp53 and RNAPII binding were identified at the gained (*MMP9* and *CCL2*) enhancers (Fig. 5b). In comparison, and consistent with pre-associated levels of mutp53 binding at the maintained enhancers, *CYP24A1*, and *CPA4*, we identified that pre-associated RNAPII levels are substantially (52% and 65%, respectively) decreased following mutp53 knockdown (Fig. 5b). Under TNF-α-induced conditions, the decrease in mutp53 binding at the enhancer regions of *MMP9* (75%, 84% at amplicons A and B, respectively), *CCL2* (87%), *CYP24A1* (74%), and *CPA4* (71%) results in a significant loss (64%, 85%, 90%, 71%, and 75%) in RNAPII binding at all four enhancers, respectively (Fig. 5b).

Consistent with the identified role of mutp53 in regulating RNAPII binding at the maintained *CYP24A1* and *CPA4* enhancers prior to TNF-α signaling, qRT-PCR analyses revealed a comparable (twofold) reduction in the uninduced eRNA and mRNA expression levels of *CYP24A1* following mutp53 knockdown (Fig. 5c). We were unable to detect a change in *CPA4* mRNA levels, which likely relates to its lower uninduced expression levels (Fig. 5c). In addition, a significant decrease in the TNF-α-induced eRNA levels from the *MMP9* (fourfold, fivefold at the −3.7 kb and −0.7 kb regions, respectively), *CCL2* (sixfold), *CYP24A1* (fivefold), and *CPA4* (fourfold) enhancers and a comparable decrease in the TNF-α-induced mRNA levels was identified for *MMP9*, *CCL2*, *CYP24A1*, and *CPA4* (eightfold, fivefold, threefold, and fourfold, respectively) following mutp53 knockdown (Fig. 5c). siRNA-mediated knockdown of mutp53 revealed an identical requirement for mutp53 in the regulation of RNAPII binding (Supplementary Fig. 5a), eRNA synthesis, and gene activation (Supplementary Fig. 5b). These findings demonstrate a strong correlation between mutp53 and RNAPII enrichment across the genome and a requirement for mutp53 in supporting RNAPII regulation of enhancer and gene activation.

To determine whether NFκB regulates TNF-inducible enhancer transcription, RNAPII ChIP-qPCR was performed in TNF-α-treated SW480 cells transfected with control or NFκB/p65 siRNA. NFκB depletion resulted in an approximately 80% decrease in RNAPII binding at the gained *MMP9* enhancers (Supplementary Fig. 5c), which is consistent with the identification that NFκB depletion reduced the levels of eRNA and mRNA *MMP9* induction by at least twofold and fivefold, respectively (Supplementary Fig. 5d). These findings are consistent with p65 regulation of mutp53 binding (Fig. 3b) and mutp53 regulation of RNAPII binding at these enhancers (Fig. 5b; Supplementary Fig. 5a). p65 also supports RNAPII binding at the maintained *CYP24A1* enhancer as revealed by an approximately 50% decrease in RNAPII levels following p65 knockdown (Supplementary Fig. 5c). However, despite the decrease in RNAPII binding, we identified little change in the TNF-α-induced eRNA levels from this enhancer (Supplementary Fig. 5d), which is likely due to the residual levels of RNAPII at this enhancer following p65 knockdown (Supplementary Fig. 5c, amplicon A versus B). In addition, TNF-α-induced *CYP24A1* mRNA levels were significantly (twofold) decreased following p65 knockdown, despite the negligible change in *CYP24A1* eRNA levels (Supplementary Fig. 5d), which suggests that p65 also regulates this target gene through mechanisms that are independent of p65 enhancer functions. Taken together, these findings demonstrate a direct requirement for the functional interplay between mutp53 and NFκB in the activation of enhancer and tumor-promoting genes.

### eRNA expression in colon carcinomas expressing mutp53

**Fig. 6:**
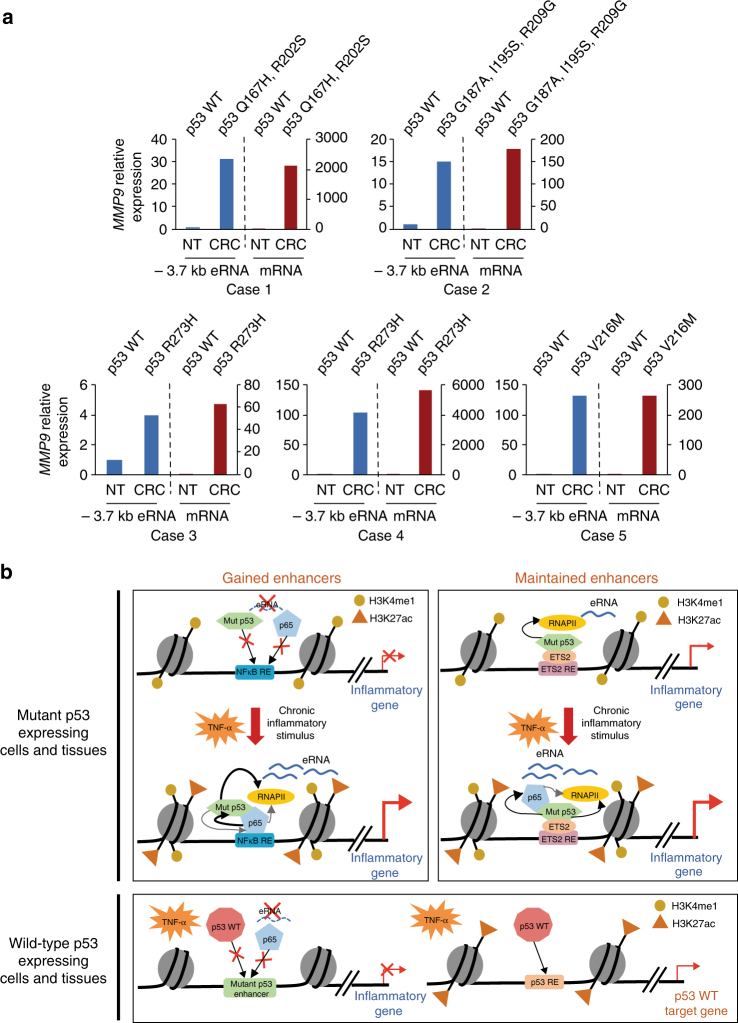
Mutp53 enhancer and gene activation in human colon tissues. **a** qRT-PCR analysis of *MMP9* eRNA from the −3.7 kb enhancer and mRNA expression levels from five independent cases of paired non-neoplastic (NT) and colorectal carcinomas (CRC) expressing wild-type and mutant forms of p53, respectively. mRNA expression levels of NT and CRC samples were normalized to β-actin levels. The expression levels shown for each CRC sample is relative to the expression levels of its corresponding NT sample. **b** Proposed model in which mutp53 through interactions with NFκB directs enhanced RNAPII recruitment that is required for the potent induction of enhancer transcription and pro-tumorigenic gene expression in response to chronic TNF signaling. The contributions of wild-type p53 were also examined in this study and are included in our model to show that the TNF-α-induced changes in gene expression are mutp53-dependent as wild-type p53 is neither recruited to the mutp53-bound enhancer regions, nor regulates enhancer activation and gene induction

To explore the clinical significance of mutp53-dependent alterations in the cancer cell transcriptome, qRT-PCR analyses were performed to analyze *MMP9* eRNA and mRNA expression levels in human colorectal carcinomas (CRCs) and matched non-neoplastic tissues isolated from five patients. First, DNA sequencing (Fig. 6a) and immunoblot (Supplementary Fig. 6a) analyses confirmed mutp53 versus wild-type p53 expression in CRCs and non-neoplastic tissues, respectively. Notably, we identified a potent activation of *MMP9* eRNA and mRNA expression levels in CRCs that express mutp53, as compared with the negligible levels that are present in the wild-type p53-expressing tissues (Fig. 6a). Additional support for these findings is the identification of significant *CCL2* eRNA and mRNA expression levels in the CRCs and not the non-neoplastic tissues (Supplementary Fig. 6b). Thus, these results further underscore the importance of mutant versus wild-type p53 in regulating the observed changes in the cancer cell transcriptome.

## Discussion

Immune cells of the tumor microenvironment influence the course of tumor progression, yet the molecular mechanisms by which immune signaling drives alterations in tumor-specific gene expression remains unclear. Our study highlights an emerging link between chronic proinflammatory signaling pathways and the gene expression landscape controlled by GOF mutations of the tumor suppressor p53. Specifically, our findings reveal that chronic TNF-α signaling prompts a global relationship between mutp53 and NFκB that supports potent enhancer and tumor-promoting gene activation and has significant implications for cancer aggressiveness. Support for a dynamic relationship between mutp53 and NFκB is demonstrated by the identification of a significant gain in the global binding of mutp53 that overlaps with NFκB at active enhancers, kinetic ChIP analyses that show a direct parallel in the temporal-binding profiles of mutp53 and NFκB, and re-ChIP analyses that revealed simultaneous binding of these factors at active enhancers.

Roles for NFκB and mutp53 in the regulation of each other’s binding at active enhancers in response to chronic TNF signaling is supported by our demonstration of (1) direct physical interactions between purified NFκB and mutp53, (2) the formation of mutp53 and NFκB associations in colon cancer cells, and (3) the decreased binding of NFκB and mutp53 at active enhancers that occurs following the depletion or inactivation of the other factor. Specifically, at the mutp53-gained enhancers, we identified that mutp53 is bound through NFκB interactions as demonstrated by the near complete loss of mutp53 binding that occurs in response to NFκB knockdown and the inhibition of NFκB activation. As the vast majority of *p53* mutations localize to the DNA-binding domain and alter the recognition of wild-type p53 sequence-specific DNA elements^28^, these findings support an additional mechanism for mutp53 DNA-binding that is facilitated by NFκB. We also identified that mutp53 knockdown leads to a modest, yet significant decrease in NFκB binding at this identical subset of enhancers. These modest effects of mutp53 depletion on NFκB binding are consistent with additional NFκB-enhancer interactions, particularly those that are likely facilitated by NFκB recognition of its consensus motifs that are present at these enhancers. The cooperativity in mutp53 and NFκB-enhancer binding is consistent with the previously described assisted loading mechanism in which factors localized at the same enhancer region facilitate each other’s binding^29^. An unexpected finding is the identification that mutp53 facilitates NFκB binding at the mutp53-maintained enhancers that are devoid of NFκB recognition sequences and that show significant and comparable levels of mutp53 and ETS2, which was previously shown to recruit mutp53^25, 26^. This finding significantly advances our understanding of the GOF properties of mutp53 by revealing the ability of mutp53 to facilitate NFκB accessing this subset of enhancers, which provides new opportunities for expanding the proinflammatory roles of NFκB in cancer cells. This observation suggests the importance of considering additional roles of mutp53 in regulating NFκB involvement in chronic inflammatory and autoimmune diseases, wherein proinflammatory cytokines drive NFκB activation. Although we are unable to rule out additional cofactors and mechanisms that may contribute to the recruitment and function of mutp53 and NFκB, our results provide strong evidence for the mutual interdependence of mutp53 and NFκB at these classes of enhancers in response to chronic TNF-α signaling.

Growing evidence supports mechanisms underlying mutp53 GOF activities that are connected to the gene regulatory roles of mutp53^30^. Yet, the full spectrum of mechanisms remains to be elucidated, and little is known about the roles of mutp53 in the regulation of enhancers. Through RNAPII ChIP-seq and GRO-seq^31^ analyses, we demonstrate that enhancer-specific mutp53 binding events are linked to RNAPII recruitment and the extensive induction of eRNA synthesis in response to chronic TNF signaling. Also, we provide a global perspective of the relationship that exists between enhancer-directed transcription and tumor-promoting gene activation mediated by the functional interplay between mutp53 and NFκB. Interestingly, the enhancers co-bound by mutp53 and NFκB support significantly higher levels of TNF-induced eRNA synthesis that positively correlates with higher levels of transcription from the nearby gene promoters as compared with the enhancers that are bound only by NFκB. We also demonstrate that mutp53 and NFκB exhibit a direct role in the regulation of RNAPII recruitment and as a result, modulate the TNF-α-inducible activation of enhancers and tumor-promoting genes. Altogether, inducible eRNA synthesis and H3K27ac accumulation are strong indicators that the induced recruitment of mutp53 and NFκB coincides with enhancer activation. This finding is consistent with previously described mechanisms in which signal-dependent transcription factors bind to pre-selected enhancers that consist of H3K4me1 and little to no enrichment of H3K27ac, and transition to an active state that includes enrichment of both H3K4me1 and H3K27ac^18^. Further investigation is needed to determine the additional factors that together with mutp53 and NFκB regulate the step-wise events that lead to enhancer activation.

Our studies also provide support for mutant versus wild-type p53 in the regulation of these enhancer-directed alterations in the cancer cell transcriptome. Although we identified comparable NFκB interactions with p53 mutants and wild-type p53, wild-type p53 was not found to overlap with NFκB at the mutp53 maintained or gained enhancers before or after TNF signaling. In addition, wild-type p53 was not found to have a role in the induction of eRNA or mRNA synthesis in response to TNF signaling. Consistent with these cell-based experiments, additional transcriptional analyses revealed increased expression of eRNA and mRNA levels of the gained target gene loci, *MMP9* and *CCL2* in solid colon tumors that express mutp53 relative to the matched normal tissues that express wild-type p53. However, we were unable to detect an increase in the eRNA and mRNA levels of the maintained genes, *CYP24A1* and *CPA4*, which we believe may relate to the lower overall eRNA and mRNA expression levels of the maintained relative to the gained gene loci. Thus, our data supporting a mutp53-specific role in enhancer regulation that is mediated by NFκB interactions is consistent with previous studies^4^ that have shown that wild-type p53 will be recruited to its typical sites within the genome consisting of wild-type p53 DNA-binding motifs (as was shown in this study by revealing wild-type p53 recruitment to its target gene, *p21*), whereas the vast majority of p53 mutants will be mislocalized to other regions in the genome based on interactions with other transcriptional regulators (i.e., NFκB in this study). Overall, the effects on enhancer and gene activation by mutp53 and NFκB enhancer landscapes in response to chronic TNF-α signaling support our proposed model (Fig. 6b), which is consistent with an emerging regulatory network of mutp53 in orchestrating chronic inflammation-induced colon cancer.

## Methods

### Cell culture and treatments

Human SW480 and HCT116 cells were purchased from American Type Culture Collection (ATCC) and grown in Dulbecco’s modified Eagle medium (DMEM, Gibco) supplemented with 10% fetal bovine serum (FBS, Gibco). MDA-MB-231 cells were also purchased from ATCC and were grown in RPMI 1640 (Gibco) medium supplemented with 10% FBS. SW480 cells that stably and inducibly express short hairpins against LacZ or p53 were kindly provided by Xinbin Chen (UC Davis) and were grown in standard DMEM medium containing 1× penicillin and streptomycin (Gemini Bio-Products), 1.5 μg ml^−1^ puromycin (Sigma), and were induced with 1 μg ml^−1^ doxycycline (Sigma). MG132 treatment experiments were performed by treating SW480 cells with 5 μM MG132 (Sigma) or vehicle (DMSO, Fisher Scientific) and with 12.5 ng ml^−1^ TNF-α (R&D Systems) for 0 or 16 h before harvesting for gene expression or ChIP analyses. All cell lines mentioned have tested negative for mycoplasma contamination by PCR.

### RNA interference experiments

SW480 and MDA-MB-231 cells were transfected with 100 nM non-targeting siRNA control, human RelA siRNA SMART pool, or p53 siRNA duplexes (listed in Supplementary Table 1) (all siRNAs are from Thermo Scientific). Forty-eight hours after transfection, cells were treated with 12.5 ng ml^−1^ TNF-α for 0 or 16 h before harvesting for gene expression or ChIP analyses.

### Immunoblotting

Protein samples were incubated at 95 °C for 5 min, separated by SDS-PAGE, and transferred to PVDF membranes (EMD Millipore) that were probed with the indicated antibodies. Reactive bands were detected by ECL (Thermo Scientific Pierce), and exposed to Blue Devil Lite ECL films (Genesee Scientific). Full scans of all western blots are provided in Supplementary Fig. 7.

### Antibodies

Antibodies used for ChIP assays were obtained commercially as followed: anti-H3 (ab1791, 1 μg), anti-H3K4me1 (ab8895, 1 μg), and anti-H3K427ac (ab4729, 1 μg) from Abcam; anti-RNA Pol II (N20, sc899, 2 μg), anti-p53 (FL393, sc6243, 2 μg), anti-ETS2 (sc351, 2 μg), anti-p65 (sc372x, 2 μg), and anti-IgG (sc2027, 2 μg) from Santa Cruz Biotechnology. Antibodies used for immunoblotting were obtained as followed: anti-p53 (DO1, sc126, 1:2000 dilution) and anti-β-Actin (sc47778, 1:2000 dilution) from Santa Cruz Biotechnology, and anti-p65 (ab7970, 1:1000 dilution) from Abcam.

### RNA purification and quantitative real-time PCR

Total RNA was extracted with TRIzol LS reagent (Invitrogen) from SW480, MDA-MB-231, HCT116, and SW480 stably expressing LacZ or p53 shRNA and treated with 12.5 ng ml^−1^ TNF-α for the indicated time points. Reactions were performed using SYBR Green PCR Master Mix (Applied Biosystems) in duplicate using samples from at least three independent cell harvests and the specificity of amplification was examined by melting curve analysis. Primers used for qRT-PCR analysis are listed in Supplementary Table 3. The relative levels of eRNA and mRNA expression were calculated according to the (ΔΔCt) method^32^ and individual expression data was normalized to GAPDH. The gene expression levels determined after TNF-α treatment are relative to the levels before TNF-α treatment.

### Invasion assay

Invasion assays were performed using SW480 cells that inducibly express shRNA against LacZ or mutp53 and were pre-treated with or without 12.5 ng ml^−1^ recombinant TNF-α in low-serum media (0.1% serum) for 24 h prior to plating 1–2 × 10^5^ cells from each condition on 24-well-PET inserts with 8 μm pore size (Falcon), coated with BD Matrigel (BD Bioscience). The lower chamber was filled with high-serum media (20% serum) without TNF-α. Cells that passed through the Matrigel after 24h were fixed, stained, imaged, and counted after. Representative scale bars were added using ImageJ.

### Colorectal carcinoma tumor analysis

Five colorectal carcinoma tumors and their corresponding paired normal colon tissue were obtained from Biorepository Tissue Technology Shared Resource at Moores Cancer Center (UC San Diego), following informed consent from patients. Samples were dissected by pathologists, and frozen in liquid nitrogen for molecular analyses. Briefly, RNA extraction from the tissue samples was done using TRIzol LS reagent (Invitrogen). Extracted RNA was subsequently treated with DNase I (Worthington Biochem) before being reverse-transcribed with the SuperScript III First-Strand Synthesis System (Invitrogen), and amplified with SYBR Green PCR master mix. qRT-PCR primer sequences can be found in Supplementary Table 3. The DNA-binding domain of p53 was amplified from cDNA by PCR and submitted for sequencing. Primers used for PCR amplification are listed in Supplementary Table 4. For extraction of protein from the tumor samples, genomic DNA was first precipitated with ethanol and isolated by centrifuging the organic phase after TRIzol purification of the RNA. Isopropanol was added to the phenol–ethanol supernatant obtained after isolation of the genomic DNA to precipitate the protein. The protein precipitate was washed with 0.3 M guanidine hydrochloride in 95% ethanol and then dissolved in 1% SDS at 50 °C. Extracted proteins were quantified using Protein Assay Reagent Dye Concentrate (Bio-Rad) and 20 µg of total protein was analyzed by immunoblot.

### Purification of recombinant proteins

GOF p53 mutants were generated by site-directed mutagenesis (Agilent Technologies). Wild-type and GOF p53 mutants were expressed in bacteria and purified on M2 agarose (Sigma). The p65 protein, which was kindly provided by Gourisankar Ghosh (UC San Diego) was purified on Ni-NTA beads (Qiagen) and Superdex 200 (GE Healthcare) columns following expression from pFAST-BAC1 vector in Sf9 cells.

### In vitro protein-binding assay

For binding assays, excess His-tagged p65 protein was incubated with FLAG-tagged p53 proteins in binding buffer (150 mM NaCl, 20 mM Hepes at pH 7.9, 0.1% NP40) for 2 h at 4 °C. The protein complexes were then incubated with M2 agarose for 30 min at 4 °C. Beads were washed five times with wash buffer (20 mM Tris-HCl at pH 7.9, 20% glycerol, 0.1 mM EDTA, 150 mM KCl, 0.1% NP40). Bound proteins were eluted in sample buffer and analyzed by immunoblot.

### Co-immunoprecipitation

For co-immunoprecipitation assays, SW480 cells were treated with 12.5 ng ml^−1^ TNF-α for 0 or 16 h, crosslinked in 1% formaldehyde (Sigma), harvested, and washed with PBS. The cell pellet was resuspended in RIPA lysis buffer (50 mM Tris-HCl at pH 7.9, 150 mM NaCl, 1% NP40, 0.1% SDS, 0.5% Na-deoxycholate), supplemented with protease inhibitor cocktail (Sigma) and incubated on ice for 30 min before isolating the nuclear pellet by centrifugation at 4 °C. The pellet was then further lysed by resuspension in hypotonic buffer (20 mM Hepes at pH 7.9, 1.5 mM MgCl_2_, 20 mM KCl, 25% glycerol) and high-salt buffer (20 mM Hepes at pH 7.9, 1.5 mM MgCl_2_, 800 mM KCl, 25% glycerol, 1% NP40) followed by rotation at 4 °C. Lysates were cleared by centrifugation and incubated with indicated antibodies for 2 h at 4 °C. After an additional 2 h incubation with Protein A Sepharose (Rockland Inc.), beads were washed with wash buffer (20 mM Tris-HCl at pH 7.9, 20% glycerol, 0.1 mM EDTA, 150 mM KCl, 0.1% NP40) five times and analyzed by immunoblotting.

### RNA-seq analysis

Total RNA was extracted with TRIzol LS reagent from SW480-expressing short hairpin RNA against either LacZ or p53, treated with 12.5 ng ml^−1^ TNF-α for 0 or 16 h. Strand-specific libraries from two biological replicates were generated from 1 µg total RNA, following the dUTP second strand cDNA method^33^. Briefly, RNA Isolation was subjected to two consecutive rounds of oligo (dT) enrichment using magnetic beads (New England Biolabs) followed by fragmentation. Isolated RNA species were then used for First-Strand cDNA Synthesis using SuperScript III in the presence of Actinomycin D (Invitrogen). Strand specificity was maintained by second-Strand synthesis with dUTP. The ends on the double stranded DNA were then repaired, followed by the addition of the A-tail. Adaptor ligation was performed using NEXTflex barcodes (Bioo Scientific) and Uracil-DNA-Glycosylase (UDG) treatment was performed to selectively degrade the strand marked with dUTP. The remaining strand was PCR amplified for nine cycles to generate a sequencing library. Final PCR products were then size selected to have an average size of 250 bp and purified using DNA Clean & Concentrator Kit (Zymo Research). DNA concentrations were determined using Qubit 2.0 fluorometer (Invitrogen) and pooled for sequencing. cDNA libraries were single-end sequenced (50 bp) on Illumina HiSeq 4000. Sequencing reads were mapped to hg38 human genome using STAR^34^. Gene expression levels were counted per gene model (not counting differentiated splices) and differential expression across samples were determined using edgeR^35^. Gene ontology enrichment (GO) analysis was performed using Metascape^36^.

### Chromatin immunoprecipitation

p53, RNAPII, and histone chromatin immunoprecipitation (ChIP) assays were performed using SW480, MDA-MB-231, or HCT116 cells that were (i) untreated or treated with 12.5 ng ml^−1^ TNF-α for the indicated time points or (ii) transfected with indicated siRNAs and treated with 12.5 ng ml^−1^ TNF-α for 0 or 16 h. Cells were reversibly cross-linked using a final concentration of 1% formaldehyde for 10 min at room temperature and quenched by adding glycine (Fisher Scientific) to a final concentration of 125 mM. In place of tip-sonication, isolated chromatin was fragmented to an average size of 200–600 bp with a biorupter Pico (Diagenode). Precleared chromatin was immunoprecipitated overnight at 4 °C and immunocomplexes were collected with Protein A agarose coupled with salmon sperm DNA (EMD Millipore) for 2 h at 4 °C. The immunocomplexes were washed and eluted, crosslinks were reversed at 65 °C for 4 h or overnight, and DNA was purified using DNA Clean & Concentrator Kit according to the manufacturer’s instructions. qPCR was performed using ABI Real-Time PCR machine to measure the relative amounts of ChIP DNA and results were quantified relative to inputs as detailed^37^. The levels of H3K4me1 and H3K27ac were determined relative to the total H3 levels. Primer sets are listed in Supplementary Table 2.

### p65 ChIP and ChIP-seq

For p65 ChIP-qPCR and all ChIP-seq assays (p53, p65, RNAPII, H3K4me1, and H3K27ac), 20–24 million cells were first crosslinked in 6 mM disuccinimidyl glutarate (ProteoChem) in PBS for 30 min, then subsequently in 1% formaldehyde in PBS for 10 min at room temperature. Crosslinking was then quenched by addition of glycine to a final concentration of 125 mM. Cells were then resuspended in lysis buffer (20 mM Tris-HCl pH 7.5, 300 mM NaCl, 2 mM EDTA, 0.5% NP40, 1% Triton X-100) and incubated on ice for 30 min. The resuspended cells were then transferred to an ice-cold homogenizer and dounced for 10 strokes. Nuclei were collected and resuspended in shearing buffer (0.1% SDS, 0.5% N-lauroylsarcosine, 1% Triton X-100, 10 mM Tris-HCl pH 8.1, 100 mM NaCl, 1 mM EDTA) and the isolated chromatin was fragmented to an average size of 200–600 bp with biorupter Pico sonicator. Immunocomplexes were collected from 65 μg of sheared chromatin with Protein A Dynabeads (Invitrogen) overnight at 4 °C. Following the overnight incubation, immunocomplexes bound to Dynabeads were resuspended in wash buffer (50 mM Hepes at pH 7.6, 500 mM LiCl, 1 mM EDTA, 1% NP40, 0.7% Na-deoxycholate). The beads were washed eight times followed by two additional TE (1× TE at pH 8, 50 mM NaCl) washes. The immunocomplexes were eluted in elution buffer (50 mM Tris-HCl at pH 8, 10 mM EDTA, 1% SDS) and the crosslinks were reversed overnight at 65 °C. Samples were subsequently treated with RNase A at 37 °C for 1 h and 0.2 µg ml^−1^ proteinase K for 2 h. The ChIP DNA was isolated using the DNA Clean & Concentrator Kit according to manufacturer’s instructions. qPCR was performed to measure the relative amounts of ChIP DNA and results were quantified relative to inputs as detailed^37^.

Sequential ChIP experiments were performed exactly as described above with minor modifications. Specifically, 150 μg of sheared chromatin was used to perform the IP. Following the washes after the first IP, immunocomplexes were eluted in re-IP elution buffer (50 mM Tris-HCl at pH 8, 1% SDS, 1 mM EDTA, 1 mM DTT), diluted 10-fold in dilution buffer (16.7 mM Tris-HCl at pH 8, 167 mM NaCl, 0.01% SDS, 1% Triton X-100, 1.2 mM EDTA), and incubated with the second IP antibodies overnight at 4 °C, followed by additional washes and the final elution as described above.

For ChIP-seq experiments, the IP’s were performed as described above and the eluted ChIP DNA was quantified using a Qubit 2.0 fluorometer, and 2–5 ng of ChIP DNA was used to prepare the sequencing libraries from two biological replicates using the TruSeq ChIP Sample Prep Kit according to the manufacturer’s instructions (Illumina). Briefly, ChIP DNA was end-repaired and Illumina TruSeq adaptors were ligated to the ends of the ChIP fragments. Adaptor-ligated ChIP DNA fragments with average size of 350 bp were used to construct libraries according to Illumina’s specifications. Prepared libraries were single-end sequenced (50 bp) on Illumina HiSeq 4000. Sequencing reads were mapped to the hg38 human genome using Bowtie2 software^38^ and default parameters. The mapped reads were then processed to make TagDirectory module using HOMER^11^ for filtering. Briefly, PCR duplications were removed and only uniquely mapped reads were kept for further analysis. The genome browser files for the resulting reads were generated by using makeUCSCfile module from HOMER. Enriched regions for p65 or histone-modification deposition were called using findPeaks module from HOMER by using preset options, factor or histone styles, respectively, and compared with the corresponding inputs. For p53 R273H peak calling, p53-enriched regions were first generated by HOMER in comparison to corresponding inputs. The resulting regions were split into subpeaks using PeakSplitter (http://www.ebi.ac.uk/research/bertone/software). For the identification of mutp53 and p65 peak colocalization, p65 peaks were first divided into two groups with and without uninduced mutp53 binding by using the co-bound option of the mergePeak module from HOMER. Induced mutp53 peaks that were co-bound with p65 with and without 0 h p53 peaks identified in the previous step were defined as maintained and gained p53 peaks. Deeptools were used to generate heat maps^39^. De novo motif analysis was performed from the top peaks, which were rank-ordered by the intensity of mutp53 and NFκB/p65 peaks and grouped as described above for defining maintained and gained mutp53 peaks using “findMotifsGenome.pl” of Homer with ±100 bp window relative to the peak center. Putative motif loci of motifs from each category (gained versus maintained) were extracted from merged mutp53 and NFκB peaks with de novo motifs using “annotatePeaks.pl”. The length of the motifs was adjusted and merged to one bed file using “intersectBed”, and motif consensus sequence logos were generated by the “seqLogo” package of R.

### Global run on-seq

Global run-on reactions were performed using SW480 cells treated with 12.5 ng ml^−1^ TNF-α for 0 or 16 h. 5 million nuclei in 100 µl freezing buffer (40% glycerol, 5 mM MgCl_2_, 0.1 mM EDTA, 50 mM Tris-HCl pH 7.8) were run on by addition of 50 µl NRO-reaction buffer (15 mM Tris-HCl pH 8, 500 mM KCl, 7.5 mM MgCl_2_, 1.5% Sarkosyl, 1.5 mM DTT, 0.2 U μl^−1^ SUPERase-in (ThermoFisher Scientific), 375 µM ATP, 375 µM GTP, 0.6 µM CTP, 375 µM BrUTP (Sigma) for 5 min at 30 °C. Reactions were stopped by addition of 750 µl TRIzol LS and purified following manufacturer’s instructions. RNA was fragmented in 10 mM Tris-HCl pH 7.5, 10 mM ZnCl_2_, 0.05% Tween 20 at 70 °C for 15 min and stopped by addition of 2× EDTA. For nascent RNA enrichment, fragmented RNA samples were incubated with 50 µl equilibrated Anti-BrdU agarose beads (Santa Cruz Biotechnology) in 500 µl GRO-binding buffer (0.25× SSPE, 0.05% Tween, 37.5 mM NaCl, 1 mM EDTA) at 4 °C for 1 h under gentle rotation. Anti-BrdU beads were equilibrated by washing once with GRO-binding buffer, followed by one wash with binding buffer with 500 mM NaCl and two consecutives washes with GRO-binding buffer. Following the IP, beads were transferred to Ultrafree MC column (EMD Millipore) and spun at 1000 rcf for 30 s. Flow through was discarded and beads were washed three times with GRO-binding buffer for 5 min. The columns were then moved to fresh tubes and RNA samples were eluted twice with 200 µl TRIzol LS under gentle shaking for 3–5 min. RNA repair and libraries were prepared from two biological replicates. Libraries were amplified for 12 cycles, size selected for 165–215 bp, and sequenced on Illumina HiSeq 4000. Reads were then mapped to hg38 genome using bowtie2. Only uniquely mapped reads were kept and at most three reads at each unique genomic position. GRO-seq reads were counted 1000 bp around specified transcription factors peaks and normalized to 10 million total reads using default setting of annotatePeaks module of HOMER. Five reads (RPKM > 0.5) were used as the threshold for defining significant eRNA synthesis.

### Data availability

All sequencing data that support the findings of this study have been deposited in the National Center for Biotechnology Information Gene Expression Omnibus (GEO) and are accessible through GEO Series Accession Number GSE102796. All other relevant data are available from the corresponding author upon reasonable request.

## Electronic supplementary material

Supplementary Information

Description of Additional Supplementary Files

Supplementary Data 1
