# Supplementary material for: Mutant p53 shapes the enhancer landscape of cancer cells in response to chronic immune signaling
Source: Nat Commun. 2017 Sep 29;8:754. doi: 10.1038/s41467-017-01117-y (PMC5622043; doi:10.1038/s41467-017-01117-y)
Supplement: Supplementary file 1 — Supplementary Information [file 41467_2017_1117_MOESM1_ESM.pdf]

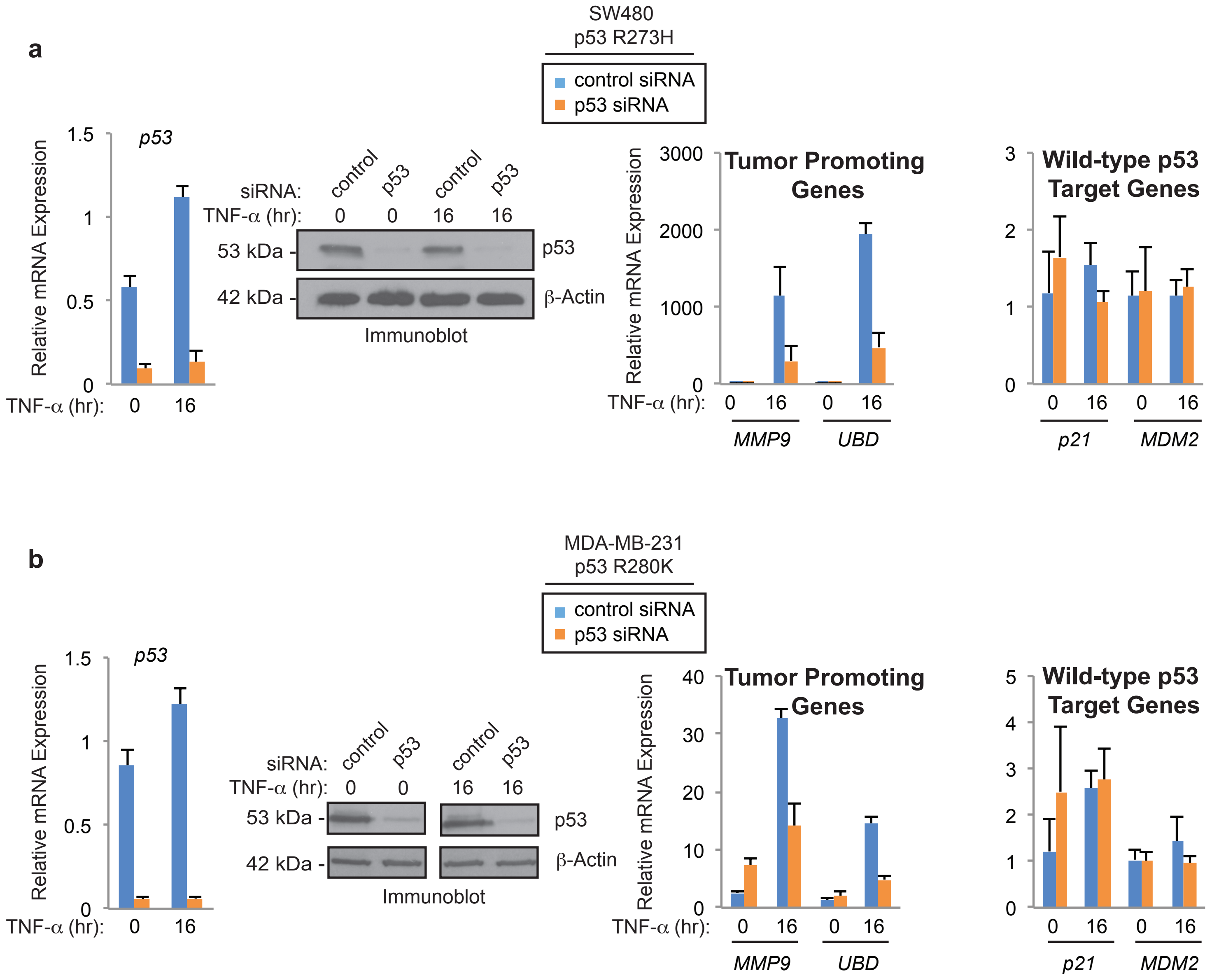
a
SW480
p53 R273H
control siRNA
p53 siRNA
p53
Relative mRNA Expression
TNF-α (hr): 0 16
siRNA: control p53 control p53
TNF-α (hr): 0 0 16 16
53 kDa
42 kDa
p53
β-Actin
Immunoblot
Tumor Promoting Genes
MMP9
UBD
Wild-type p53 Target Genes
p21
MDM2
b
MDA-MB-231
p53 R280K
control siRNA
p53 siRNA
p53
Relative mRNA Expression
TNF-α (hr): 0 16
siRNA: control p53 control p53
TNF-α (hr): 0 0 16 16
53 kDa
42 kDa
p53
β-Actin
Immunoblot
Tumor Promoting Genes
MMP9
UBD
Wild-type p53 Target Genes
p21
MDM2

**Supplementary Figure 1. Mutp53 Knockdown Disrupts TNF-α-Induced Gene Expression in Colon and Breast Cancer Cells, Related to Figure 1 (Left)** qRT-PCR and immunoblot analysis of **(a)** SW480 cells or **(b)** MDA-MB-231 cells transfected with nonspecific control or p53 siRNAs and treated with TNF-α for 0 or 16 hr. **(Right)** qRT-PCR analyses to examine the relative mRNA expression levels of mutp53 and wild-type p53 target genes in SW480 and MDA-MB-231 cells treated as described above. The expression levels upon TNF-α treatment are relative to the levels before treatment. The bar graphs represent the average of three independent experiments with the error bars denoting the standard error.

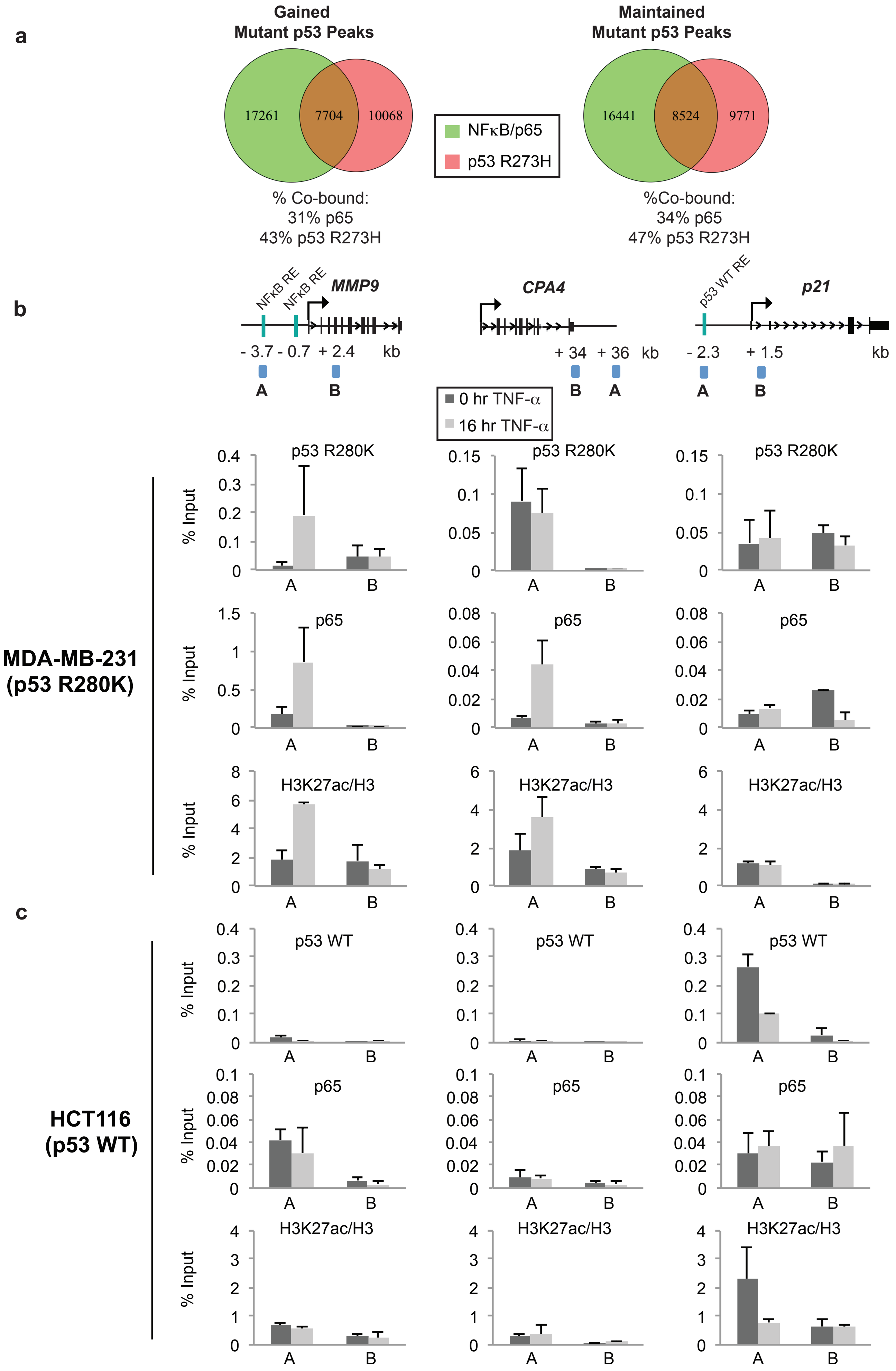
a
Gained
Mutant p53 Peaks
17261
7704
10068
% Co-bound:
31% p65
43% p53 R273H
NFκB/p65
p53 R273H
Maintained
Mutant p53 Peaks
16441
8524
9771
%Co-bound:
34% p65
47% p53 R273H
b
NFκB RE
NFκB RE
MMP9
- 3.7
- 0.7
+ 2.4
kb
A
B
CPA4
+ 34
+ 36
kb
B
A
p53 WT RE
p21
- 2.3
+ 1.5
kb
A
B
0 hr TNF-α
16 hr TNF-α
MDA-MB-231
(p53 R280K)
p53 R280K
p65
H3K27ac/H3
% Input
c
HCT116
(p53 WT)
p53 WT
p65
H3K27ac/H3
% Input

**Supplementary Figure 2. p53 and NFκB binding upon Chronic TNF-α Signaling in mutp53 Expressing Breast and Wild-type p53 Expressing Colon Cancer Cells, Related to Figure 2 (a)** Venn diagram showing the overlap of NFκB/p65 with the gained or maintained mutp53 binding sites after 16 hr TNF-α. **(b)** ChIP analyses with the indicated antibodies were performed using MDA-MB-231 (p53 R280K) or **(c)** HCT116 (p53 WT) cells that were treated with TNF-α for 0 or 16 hr. The amplicons used for ChIP-qPCR are represented in the schematics of the target gene loci. Also, the amplicon for the wild-type p53 target gene, *p21* was designed to amplify the region of *p21* that overlaps with a wild-type p53 response element. ChIPs for H3K27ac were normalized to H3. An average of two independent ChIP experiments that are representative of at least three is shown with error bars denoting the standard error.

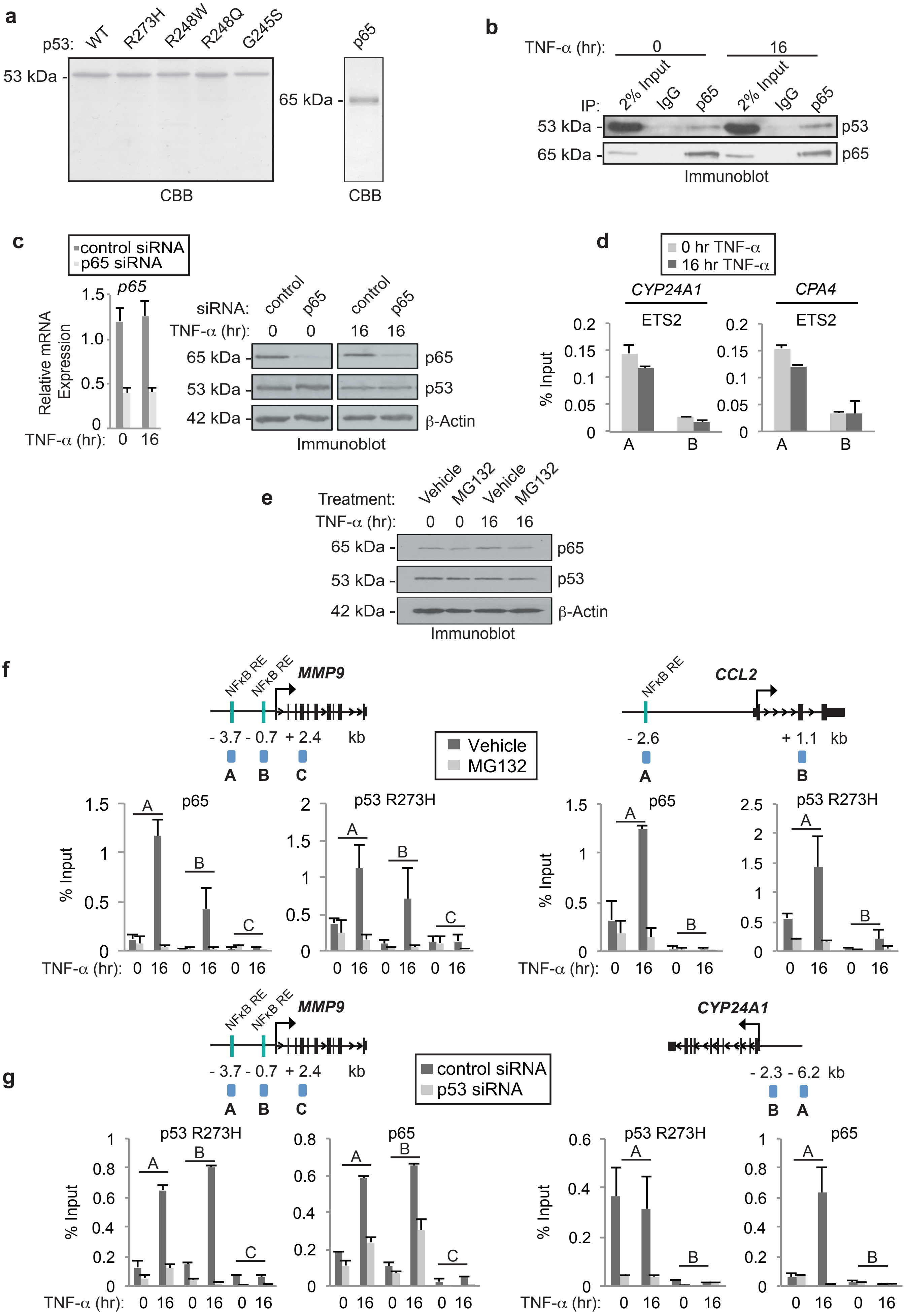
a
p53: WT R273H R248W R248Q G245S
53 kDa
CBB
p65
65 kDa
CBB
b
TNF-α (hr): 0 16
IP: 2% Input IgG p65 2% Input IgG p65
53 kDa p53
65 kDa p65
Immunoblot
c
control siRNA
p65 siRNA
p65
Relative mRNA Expression
1.5
1.0
0.5
0
TNF-α (hr): 0 16
siRNA: control p65 control p65
TNF-α (hr): 0 0 16 16
65 kDa p65
53 kDa p53
42 kDa β-Actin
Immunoblot
d
0 hr TNF-α
16 hr TNF-α
CYP24A1
CPA4
ETS2
% Input
A B
e
Treatment: Vehicle MG132 Vehicle MG132
TNF-α (hr): 0 0 16 16
65 kDa p65
53 kDa p53
42 kDa β-Actin
Immunoblot
f
NFκB RE
MMP9
- 3.7 - 0.7 + 2.4 kb
A B C
Vehicle
MG132
CCL2
- 2.6 + 1.1 kb
p65
p53 R273H
% Input
TNF-α (hr): 0 16 0 16 0 16
g
MMP9
control siRNA
p53 siRNA
CYP24A1
- 2.3 - 6.2 kb
p53 R273H
p65
% Input
TNF-α (hr): 0 16 0 16 0 16

**Supplementary Figure 3. Analysis of NFκB and Mutp53 at Proinflammatory Target Gene Enhancers, Related to Figure 3 (a)** Analysis of purified p53 and p65 proteins by SDS-PAGE with Coomassie Brilliant Blue (CBB) staining. **(b)** Immunoblot analyses following p65 Co-IP performed with nuclear extract from SW480 cells treated with TNF-α for 0 or 16 hr with indicated antibodies that recognize mutp53 and p65. Three independent Co-IP assays were performed. **(c)** qRT-PCR and Immunoblot analysis of SW480 cells that were transfected with non-targeting control or NFκB/p65 siRNA, following TNF-α treatment for 0 or 16 hr with indicated antibodies. The expression levels upon TNF-α treatment are relative to the levels before treatment. The bar graphs represent the average of three independent experiments with the error bars denoting the standard error. **(d)** ETS2 ChIP-qPCR analysis in SW480 cells following TNF-α treatment for 0 or 16 hr at *CYP24A1* and *CPA4* enhancer and nonspecific regions. The amplicons used for ChIP-qPCR are shown in the schematics of the target gene loci. **(e)** Immunoblot analysis of SW480 cells treated with vehicle or MG132 and TNF-α for 0 or 16 hr with indicated antibodies. **(f)** ChIP analyses to examine NFκB/p65 and mutp53 binding at the enhancers and nonspecific regions of *MMP9* and *CCL2* in SW480 cells treated with vehicle or MG132 and TNF-α for the indicated time points. The amplicons used for ChIP-qPCR are shown in the schematics of the target gene loci. **(g)** ChIP analyses of p53 R273H and NFκB/p65 binding at the enhancers and nonspecific regions of *MMP9* and *CYP24A1* in SW480 cells transfected with control or p53 siRNA and treated with TNF-α for the indicated time points. The amplicons used for ChIP-qPCR are shown in the schematics of the target gene loci. For all ChIP assays, an average of two independent ChIP experiments that are representative of at least three is shown with error bars denoting the standard error.

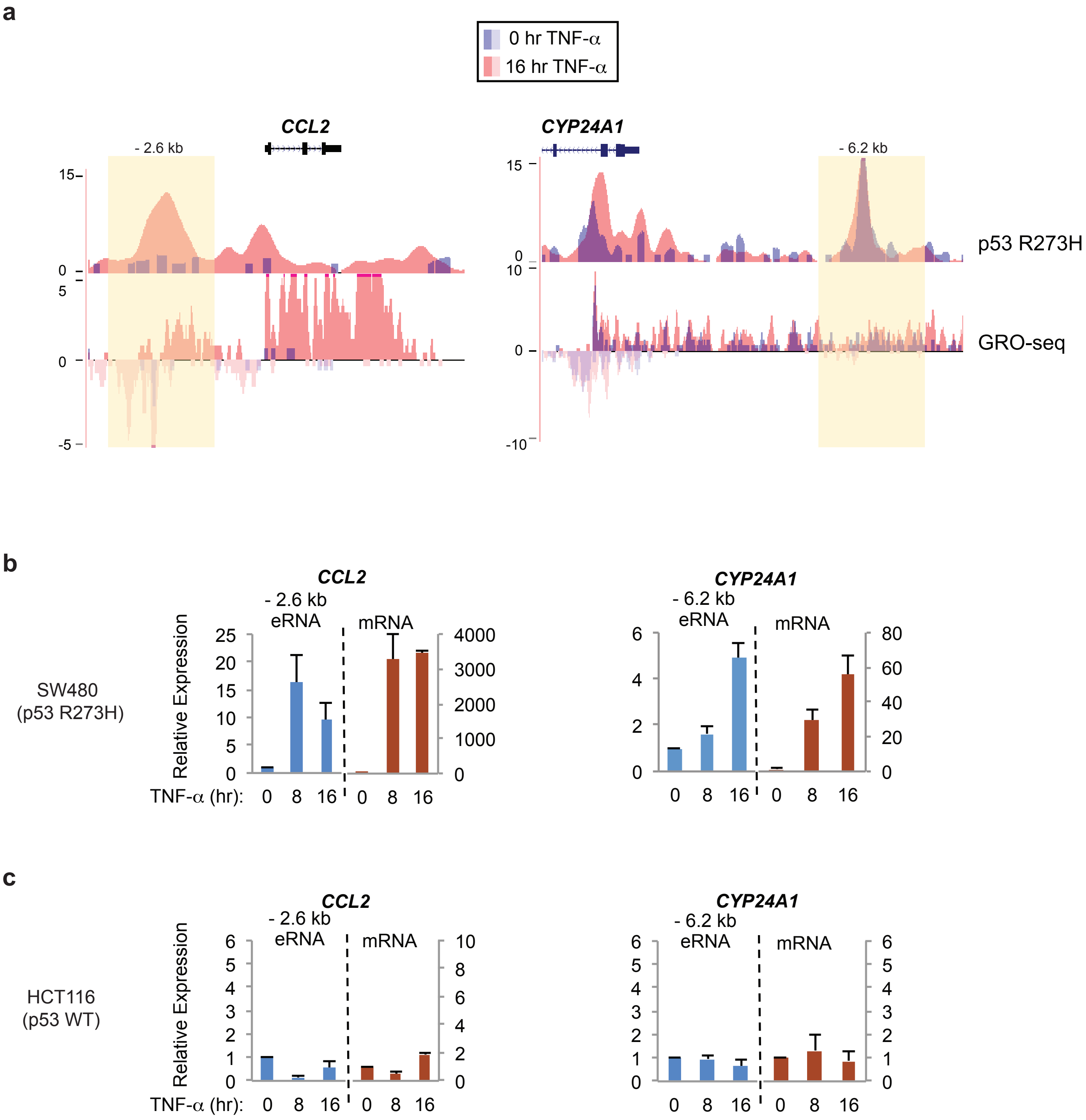

a
0 hr TNF-α
16 hr TNF-α
CCL2
- 2.6 kb
CYP24A1
- 6.2 kb
p53 R273H
GRO-seq
b
SW480
(p53 R273H)
CCL2
- 2.6 kb
eRNA
mRNA
CYP24A1
- 6.2 kb
eRNA
mRNA
Relative Expression
TNF-α (hr):
c
HCT116
(p53 WT)
CCL2
- 2.6 kb
eRNA
mRNA
CYP24A1
- 6.2 kb
eRNA
mRNA
Relative Expression
TNF-α (hr):

**Supplementary Figure 4. Mutp53 Enhancer Binding is Positively Correlated with Enhancer Transcription and Gene Activation, Related to Figure 4 (a)** UCSC genome browser images of genomic loci for *CCL2* and *CYP24A1* showing the ChIP-seq signal for mutp53 ChIP-seq and GRO-seq peaks with the enhancer regions highlighted in yellow. **(b)** qRT-PCR analysis of the indicated eRNAs and mRNAs in SW480 cells (p53 R273H), and **(c)** HCT116 cells (p53 WT) treated with TNF-α for 0, 8 or 16 hr. The expression levels shown after TNF-α treatment are relative to the levels before treatment. The bar graphs represent the average of three independent experiments with the error bars denoting the standard error.

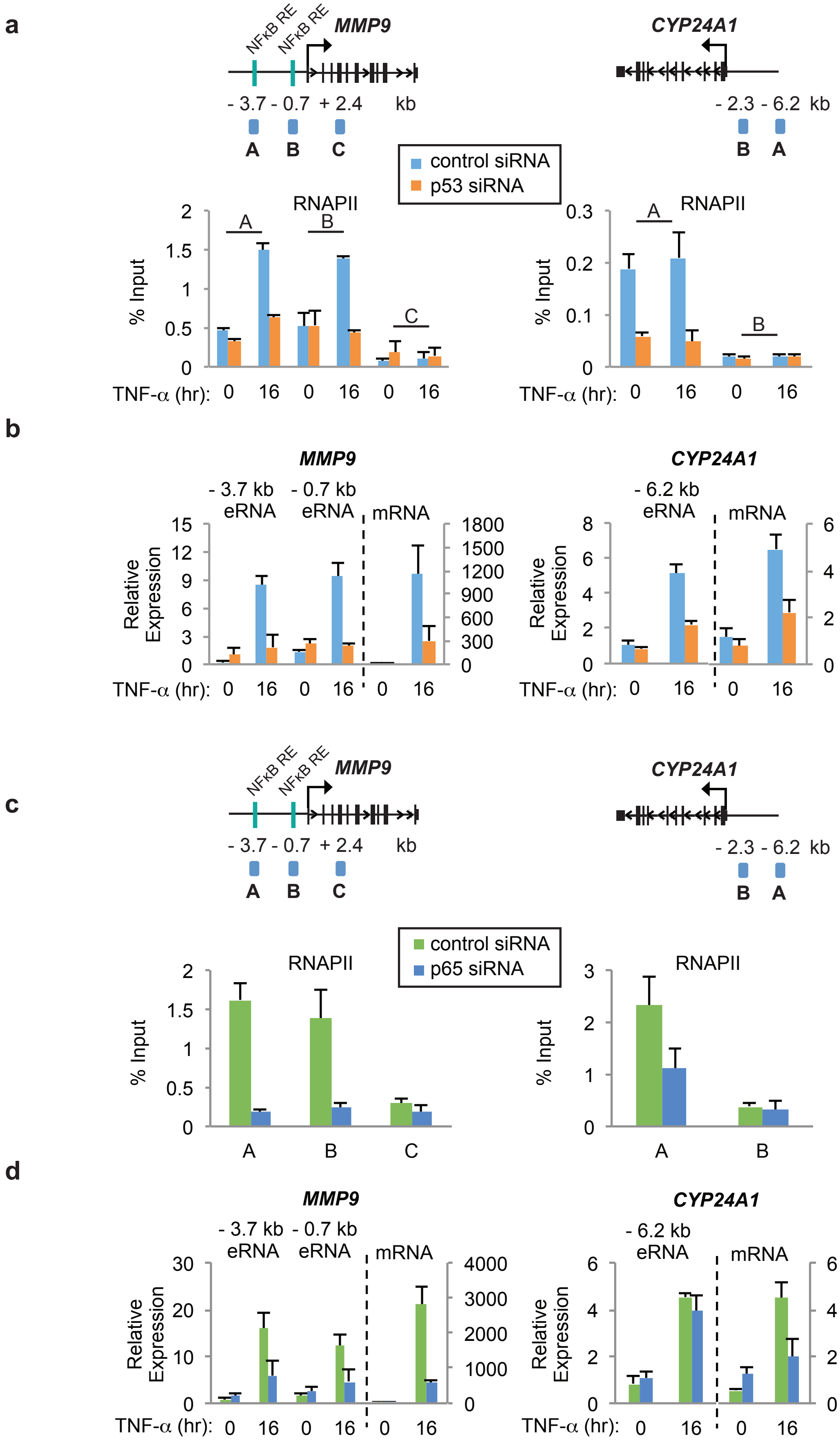
a
NFκB RE
NFκB RE
MMP9
- 3.7 - 0.7 + 2.4 kb
A B C
CYP24A1
- 2.3 - 6.2 kb
B A
control siRNA
p53 siRNA
RNAPII
% Input
TNF-α (hr): 0 16 0 16 0 16
RNAPII
% Input
TNF-α (hr): 0 16 0 16
b
MMP9
- 3.7 kb eRNA
- 0.7 kb eRNA
mRNA
Relative Expression
TNF-α (hr): 0 16 0 16 0 16
CYP24A1
- 6.2 kb eRNA
mRNA
Relative Expression
TNF-α (hr): 0 16 0 16
c
NFκB RE
NFκB RE
MMP9
- 3.7 - 0.7 + 2.4 kb
A B C
CYP24A1
- 2.3 - 6.2 kb
B A
control siRNA
p65 siRNA
RNAPII
% Input
A B C
RNAPII
% Input
A B
d
MMP9
- 3.7 kb eRNA
- 0.7 kb eRNA
mRNA
Relative Expression
TNF-α (hr): 0 16 0 16 0 16
CYP24A1
- 6.2 kb eRNA
mRNA
Relative Expression
TNF-α (hr): 0 16 0 16

**Supplementary Figure 5. Regulation of RNAPII Recruitment, eRNA Synthesis and mRNA Expression by Mutp53 and NFκB, Related to Figure 5 (a)** ChIP-qPCR analyses of RNAPII binding at the enhancers and nonspecific regions of *MMP9* and *CYP24A1* in SW480 cells transfected with control or p53 siRNA and treated with TNF-α for 0 or 16 hr. The amplicons used for ChIP-qPCR are shown in the schematics of the target gene loci. An average of two independent ChIP experiments is shown with error bars denoting the standard error. See Supplementary Fig. 1 and 3 for the relative mRNA, protein levels of mutp53, and mutp53 recruitment following p53 siRNA-mediated knockdown in SW480 cells. **(b)** qRT-PCR analysis of the *MMP9* and *CYP24A1* eRNAs and mRNAs in SW480 cells treated as described in (a). The expression levels shown after TNF-α treatment are relative to the levels before treatment. The bar graphs represent the average of three independent experiments with the error bars denoting the standard error. **(c)** ChIP-qPCR analyses of RNAPII binding at the enhancers and nonspecific regions of *MMP9* and *CYP24A1* in SW480 cells transfected with control or NFκB/p65 siRNA and treated with TNF-α for 16hr. The amplicons used for ChIP-qPCR are shown in the schematics of the target gene loci. An average of two independent ChIP experiments that are representative of at least three is shown with error bars denoting the standard error. See Supplementary Fig. 3 and Fig. 3 for NFκB/p65 mRNA, protein levels, and recruitment following NFκB/p65 siRNA-mediated knockdown in SW480 cells. **(d)** qRT-PCR analysis of the *MMP9* and *CYP24A1* eRNAs and mRNAs in SW480 cells treated as described in (c). The expression levels shown after TNF-α treatment are relative to the levels before treatment. The bar graphs represent the average of three independent experiments with the error bars denoting the standard error.

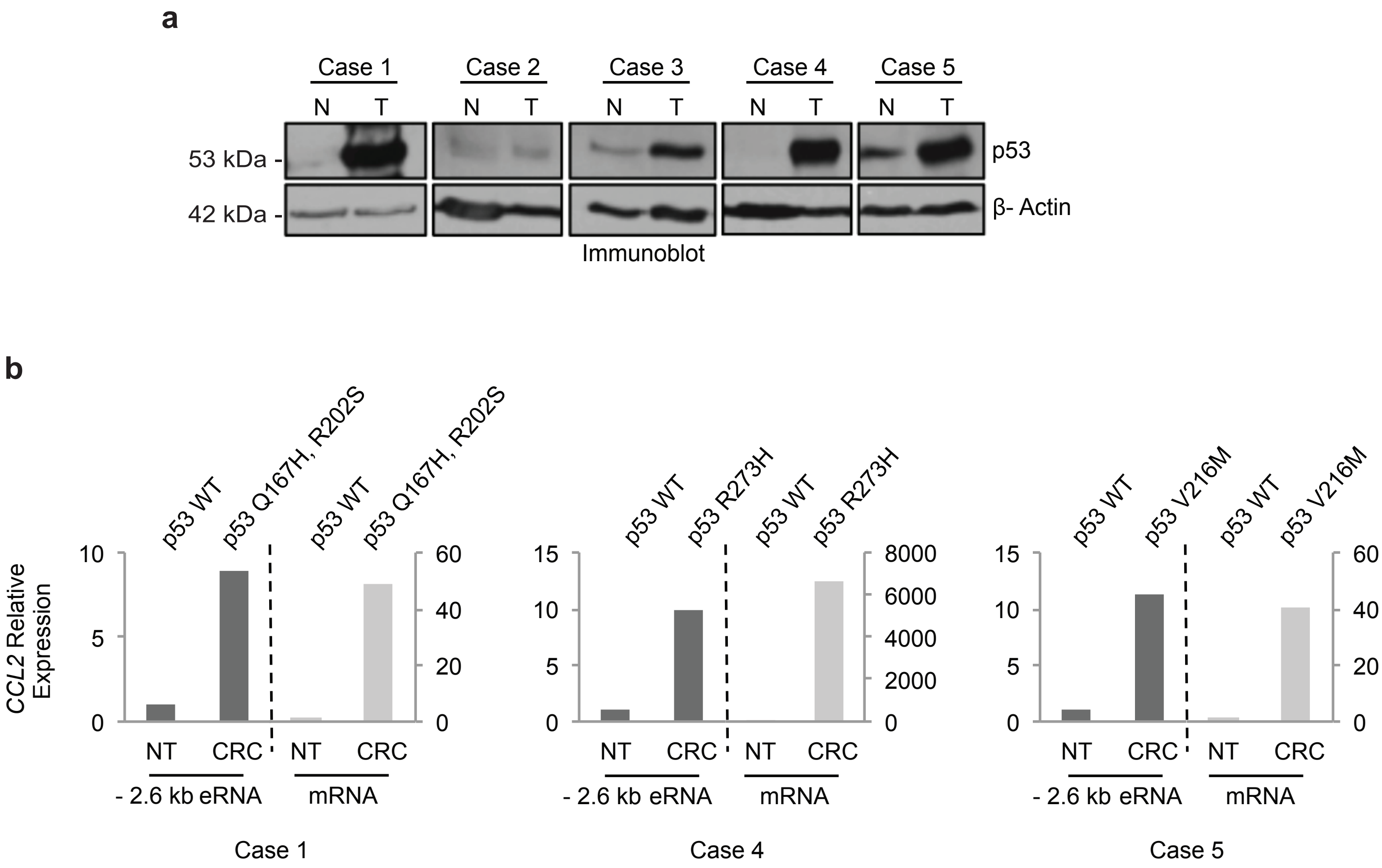
a
Case 1
Case 2
Case 3
Case 4
Case 5
N T
53 kDa -
42 kDa -
p53
β- Actin
Immunoblot
b
p53 WT
p53 Q167H, R202S
p53 R273H
p53 V216M
*CCL2* Relative Expression
NT
CRC
- 2.6 kb eRNA
mRNA
Case 1
Case 4
Case 5

**Supplementary Figure 6. eRNA and mRNA Expression Analysis in Human Colon Cancer Tissues, Related to Figure 6 (a)** Immunoblot analysis of whole lysates derived from the five cases of paired non-neoplastic (NT) and colorectal carcinomas (CRC) samples with indicated antibodies. **(b)** qRT-PCR analysis of *CCL2* - 2.6 kb eRNA and mRNA from three of the five independent cases of paired NT and CRC samples prepared and analyzed as described in Figure 6a.

**a**

shRNA-mediated p53 knockdown in SW480 cells, corresponding to Figure 1a:

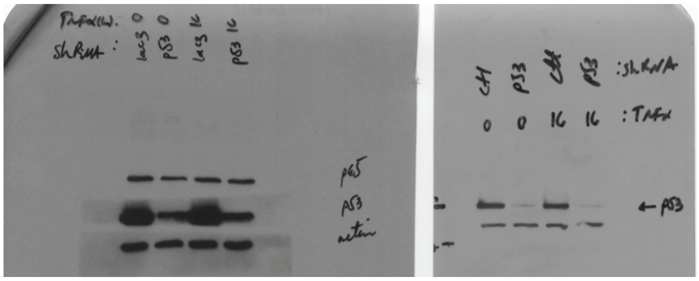

**b**

siRNA-mediated p53 knockdown in SW480 cells, corresponding to Supplementary Figure 1a:

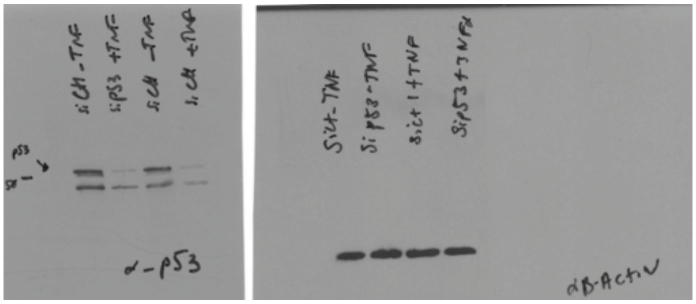

**c**

siRNA-mediated p53 knockdown in MDA-MB-231 cells, corresponding to Supplementary Figure 1b:

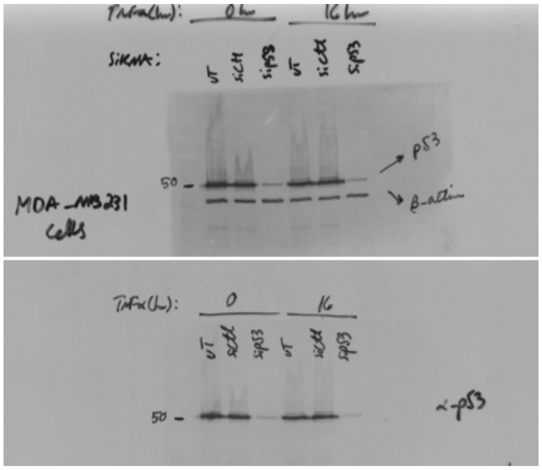

**d**

Direct p53-p65 interaction assay, corresponding to Figure 3a

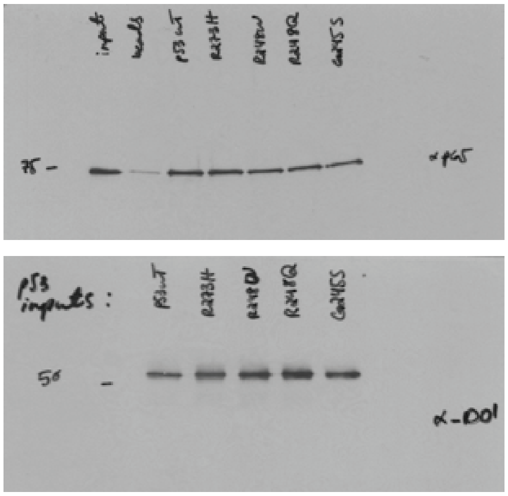

**e**

siRNA-mediated p65 knockdown in SW480 cells, corresponding to Supplementary Figure 3c:

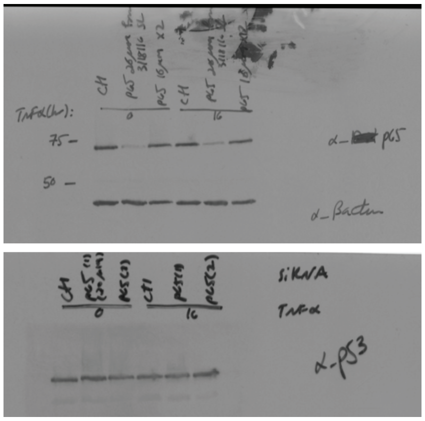

**f**

MG132 treatment of SW480 cells, corresponding to Supplementary Figure 3d:

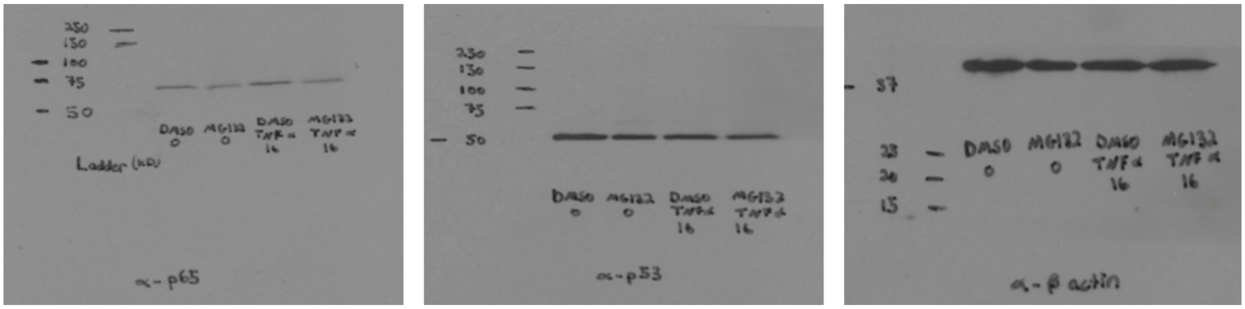

**Supplementary Figure 7. Uncropped images of western blot figures shown in the main paper**

**Supplementary Table 1. p53 and Rel-A siRNA sequences, Related to Experimental Procedures**

| siRNA | Sequence |
|---|---|
| Individual siGENOME TP53 | GCUUCGAGAUGUUCCGAGA |
| SMART pool siGENOME RELA | GGAUUGAGGAGAAACGUAA<br>CUCAAGAUCUGCCGAGUGA<br>GGCUAUAACUCGCCUAGUG<br>GAUUGAGGAGAAACGUAAA |

**Supplementary Table 2. Oligonucleotide Sequences for ChIP Analysis of the *MMP9*, *CCL2*, *CYP24A1, CPA4,* and *CDKN1A* Genes Related to Experimental Procedures**

| Region | Forward Primer | Reverse Primer |
|---|---|---|
| *MMP9* A | AGGGTCTTGGCTAAACTCTATT | TGGACAGAGCTTGGCTTTC |
| *MMP9* B | CCCTTTACTGCCCTGAAGATT | TTCTTTGACTCAGCTTCCTCTC |
| *MMP9* C | GATGGTCCTGGGTTCTAATTCC | GCAGTTCATCCCATCTCTCATC |
| *CCL2* A | TTTGTGCCAGAGCCTAACC | AGTTCCCAGATCCCGTAGAA |
| *CCL2* B | CAAAGAAGCTGTGATGTGAGTTC | GCACTCTCTGACTCTAGGTTTATG |
| *CYP24A1 A* | TACGCAGTCTTTGTGCAGTAG | GGAGGTTACATCGCTGTTCTC |
| *CYP24A1 B* | AATGCCTACCATGTCAGTATGT | CTACACTCAGCCAGAGCTATTC |
| *CPA4 A* | TTCTTTCTCTGGGAGCTTTCC | GTTTGGAAGCTGGACCTATGA |
| *CPA4 B* | GGGCAATCATAGCTCACTGTAG | TGCCTGTAATCCCAGCATTT |
| *CDKN1A A* | AGCAGGCTGTGGCTCTGATT | CAAAATAGCCACCAGCCTCTTCT |
| *CDKN1A B* | TTTGGGCGTGGAGATAAGGTGGA | GGGCGTGTGTGTGTGTGTGT |

**Supplementary Table 3. Oligonucleotide Sequences for RT-PCR Analysis of Gene Expression, Related to the Experimental Procedures**

| Gene | Forward Primer | Reverse Primer |
|---|---|---|
| *GAPDH* | ATTTGGTCGTATTGGGCGCCTG | AGCCTTGACGGTGCCATGGAATTT |
| *β-Actin* | ACCATGTACCCTGGCATTG | TACTTGCGCTCAGGAGGA |
| *p53* | GTTTCCGTCTGGGCTTCTT | GCAGGTCTTGGCCAGTT |
| *p65* | AGCACAGATACCACCAAGAC | CGGCAGTCCTTTCCTACAA |
| *MMP9* | GACCTGGGCAGATTCCAAA | GGCAAGTCTTCCGAGTAGTTT |
| *CCL2* | CAGCCAGATGCAATCAATGCC | TGGAATCCTGAACCCACTTCT |
| *CYP24A1* | TGGAGATCAAACCGTGGAAGG | GAAGACTGGCAGCGGGT |
| *CPA4* | GTCAGAAATGGAGACGAGATCAG | CTTCAATCGGCCTGTGGAT |
| *LTB* | TTTCAGAAGCTGCCAGAGG | AAACGCCTGTTCCTTCGT |
| *UBD* | CAATGCTTCCTGCCTCTGT | TCACGCTGTCATATGGGTTG |
| *MMP9* 3.7kb eRNA | TGATGGAGCTACCTCAGTG | CCACAATAGAGTTTAGCCAAGA |
| *MMP9* 0.7kb eRNA | CCCTTTACTGCCCTGAAGATT | TTCTTTGACTCAGCTTCCTCTC |
| *CCL2* eRNA | TTGTGGAGCAAGGGACAAG | CCCTTGGGTGCCTCAGTTT |
| *CYP24A1* eRNA | TACGCAGTCTTTGTGCAGTAG | GGAGGTTACATCGCTGTTCTC |
| *CPA4* eRNA | TTCTTTCTCTGGGAGCTTTCC | GTTTGGAAGCTGGACCTATGA |
| *p21* | TGGAGACTCTCAGGGTCGAAAACGGC | AGGGCTTCCTCTTGGAGAAGATCA |
| *MDM2* | AGGGCTTCCTCTTGGAGAAGATCA | GTGCACCAACAGACTTTAATAACTTCA |
| *PTG2* | GAGTGTGGGATTTGACCAGTAT | TGTGTTTGGAGTGGGTTTCA |

**Supplementary Table 4. Oligonucleotide Sequences for Amplification of p53 from Colorectal Carcinoma Tumor Samples Prior to Sequencing**

| | Forward Primer | Reverse Primer |
|---|---|---|
| p53 DBD | CAGCTGTGGGTTGATTCC | AGGGATCCTCAGTCTGAGTCAGGCCCTT |
