# Supplementary material for: Mutant p53 shapes the enhancer landscape of cancer cells in response to chronic immune signaling
Source: Nat Commun. 2017 Sep 29;8:754. doi: 10.1038/s41467-017-01117-y (PMC5622043; doi:10.1038/s41467-017-01117-y)
Supplement: Supplementary file 2 — Description of Additional Supplementary Files [file 41467_2017_1117_MOESM2_ESM.pdf]

Description of Additional Supplementary Files

File Name: Supplementary Data 1
Description: List of the TNF-responsive genes that are differentially regulated by mutp53 and that correspond to the heat map of the RNA-seq data represented in Figure 1d.
